# Models Integrating Genetic and Lifestyle Interactions on Two Adiposity Phenotypes for Personalized Prescription of Energy-Restricted Diets With Different Macronutrient Distribution

**DOI:** 10.3389/fgene.2019.00686

**Published:** 2019-07-30

**Authors:** Omar Ramos-Lopez, Jose I. Riezu-Boj, Fermin I. Milagro, Marta Cuervo, Leticia Goni, J. Alfredo Martinez

**Affiliations:** ^1^Department of Nutrition, Food Science and Physiology, and Center for Nutrition Research, University of Navarra, Pamplona, Spain; ^2^Medical and Psychology School, Autonomous University of Baja California, Tijuana, Baja California, Mexico; ^3^Navarra Institute for Health Research (IdiSNA), Pamplona, Spain; ^4^CIBERobn, Fisiopatología de la Obesidad y la Nutrición; Carlos III Health Institute, Madrid, Spain; ^5^Madrid Institute of Advanced Studies (IMDEA Food), Madrid, Spain

**Keywords:** obesity, genetics, genetic risk score, weight loss, precision nutrition, high-protein diet, low-fat diet

## Abstract

**Aim:** To analyze the influence of genetics and interactions with environmental factors on adiposity outcomes [waist circumference reduction (WCR) and total body fat loss (TFATL)] in response to energy-restricted diets in subjects with excessive body weight.

**Materials and Methods:** Two hypocaloric diets (30% energy restriction) were prescribed to overweight/obese subjects during 16 weeks, which had different targeted macronutrient distribution: a low-fat (LF) diet (22% energy from lipids) and a moderately high-protein (MHP) diet (30% energy from proteins). At the end of the trial, a total of 201 participants (LF diet = 105; MHP diet = 96) who presented good/regular dietary adherence were genotyped for 95 single nucleotide polymorphisms (SNPs) previously associated with weight loss through next-generation sequencing from oral samples. Four unweighted (uGRS) and four weighted (wGRS) genetic risk scores were computed using statistically relevant SNPs for each outcome by diet. Predictions of WCR and TFATL by diet were modeled through recognized multiple linear regression models including genetic (single SNPs, uGRS, and wGRS), phenotypic (age, sex, and WC, or TFAT at baseline), and environment variables (physical activity level and energy intake at baselines) as well as eventual interactions between genes and environmental factors.

**Results:** Overall, 26 different SNPs were associated with differential adiposity outcomes, 9 with WCR and 17 with TFATL, most of which were specific for each dietary intervention. In addition to conventional predictors (age, sex, lifestyle, and adiposity status at baseline), the calculated uGRS/wGRS and interactions with environmental factors were major contributors of adiposity responses. Thus, variances in TFATL-LF diet, TFATL-MHP diet, WCR-LF diet, and WCR-MHP diet were predicted by approximately 38% (optimism-corrected adj. *R* ^2^ = 0.3792), 32% (optimism-corrected adj. *R* ^2^ = 0.3208), 22% (optimism-corrected adj. *R* ^2^ = 0.2208), and 21% (optimism-corrected adj. *R* ^2^ = 0.2081), respectively.

**Conclusions:** Different genetic variants and interactions with environmental factors modulate the differential individual responses to MHP and LF dietary interventions. These insights and models may help to optimize personalized nutritional strategies for modeling the prevention and management of excessive adiposity through precision nutrition approaches taking into account not only genetic information but also the lifestyle/clinical factors that interplay in addition to age and sex.

## Introduction

Overweight and obesity are physiopathological conditions characterized by excessive body fat mass that may have adverse effects on health status (World Health Organization (WHO), 2018). Increased fat accumulation, especially in the intra-abdominal region, is generally associated with a cluster of metabolic disorders including glucose intolerance, insulin resistance, chronic low-grade inflammation, dyslipidemia, and high blood pressure (Tchernof and Després, 2013). Indeed, waist circumference (WC) as a measure of abdominal obesity has been commonly used to assess cardiometabolic risk, being commonly included within the diagnostic criteria to identify individuals with features of the metabolic syndrome (Kaur, 2014). On the other hand, total body fat is an important marker of adiposity-related alterations in both sexes (World Health Organization (WHO), 2018).

Overall, the combination of excessive calorie consumption and sedentary behaviors is considered the main driver of the rapid acceleration of the obesity epidemic worldwide (Romieu et al., 2017). Therefore, comprehensive lifestyle modification programs promoting a negative energy balance through improvements in diet features and physical activity represent a first line of therapy for obesity management in primary health centers (Wadden et al., 2012). Thus, many energy-restricted diets with different macronutrient distribution (proteins, carbohydrates, and lipids) have been evaluated to achieve sustainable weight loss and positive changes in metabolic alterations in subjects with overweight or obesity (Abete et al., 2010). However, heterogeneous responses to dietary interventions are well documented, ranging from resistance to reduce body fat mass to unsuccessful long-term weight loss maintenance (Blomain et al., 2013; Ramos-Lopez et al., 2017).

To date, several trials have reported some factors modulating body weight loss including age, sex, initial body weight, and level of physical activity at baseline (Greenberg et al., 2009; Delahanty et al., 2013; Look AHEAD Research Group, 2014; Bray et al., 2017). In addition, emerging evidence suggests that genetic factors affecting appetite, energy utilization, and fat deposition may partially explain the inter-individual variability in weight loss (Goni et al., 2016). Certainly, a number of single nucleotide polymorphisms (SNPs) have been associated with diverse adiposity outcomes in response to energy-restricted diets varying in macronutrient distribution (Martínez and Milagro, 2015). More importantly, potential interactions between gene and environmental factors have been shown to modulate changes in body composition and fat distribution after following specific lifestyle programs (Heianza and Qi, 2017). Together, these scientific insights show the need to personalize dietary treatments in order to optimize weight management goals (Phillips, 2013). The aim of this study was to analyze the influence of genetics and interactions with environmental factors in two different adiposity outcomes (WC and total fat mass) in response to energy-restricted diets in subjects with excessive body weight.

## Materials and Methods

### Participants

The current randomized clinical trial (Obekit, reg. no. NCT02737267, clinicaltrials.gov) enrolled overweight or obese (BMI 25–40 kg/m^2^) Spanish adults of self-reported Caucasian ancestry who were recruited at the metabolic unit of the Center for Nutrition Research of the University of Navarra. Major exclusion criteria were a clinical history of cardiovascular disease and type 1 diabetes; type 2 diabetic patients treated with insulin; pregnant or lactating women; individuals reporting weight change (>3 kg) within the 3 months before the study; use of medication that affects body weight composition; and unstable dose of medication for hyperlipidemia and hypertension treatments. Subjects drinking a relevant amount of alcohol (>40 g of ethanol/day in men and >20 g of ethanol/day in women) were excluded from the trial. A scheme showing the study design and flow of participants throughout the trial according to the 2010 CONSORT requirements is presented (**Supplementary Figure 1**). The study protocol was approved by the Research Ethics Committee of the University of Navarra (Ref. 132/2015). The research was performed in accordance with the ethical principles of the 2013 Declaration of Helsinki (World Medical Association, 2013). Participants voluntarily provided a written informed consent after they were informed about details and procedures of the protocol.

### Anthropometric and Blood Pressure Measurements

Body weight (kg), height (cm), and WC (cm) were measured following validated methods (Lopez-Legarrea et al., 2013). Body mass index (BMI) was calculated as the ratio between body weight and squared meters (kg/m^2^). Dual-energy X-ray absorptiometry was applied to estimate total body fat (TFAT, %) and visceral fat (VFAT, kg) contents following the company instructions (DEXA, Lunar Prodigy, software version 6.0, Madison, WI, USA). Blood pressure was determined using an automated sphygmomanometer according to the standardized criteria of the World Health Organization and the International Society of Hypertension (Whitworth and Chalmers, 2004).

### Blood Tests

Fasting blood samples were drawn by venipuncture and centrifuged for serum extraction. Blood tests including glucose (mg/dl), total cholesterol (TC, mg/dl), high-density lipoprotein cholesterol (HDL-c, mg/dl), and triglycerides (TG, mg/dl) were determined in an automatic analyzer (Pentra C200, HORIBA Medical) using appropriate commercial kits. The triglyceride-glucose (TyG) index was estimated applying the following formula: TyG index = (ln [fasting triglycerides (mg/dl) × fasting plasma glucose (mg/dl)/2]) as described elsewhere (Navarro-González et al., 2016).

### Dietary Intake and Physical Activity Assessments

Food consumption during the year before the study was evaluated using a validated 137-item food frequency questionnaire (Martin-Moreno et al., 1993; de la Fuente-Arrillaga et al., 2010; Fernández-Ballart et al., 2010). Total energy (kcal) and macronutrient intakes (%) were obtained from standard Spanish food composition tables (Moreiras et al., 2013). Physical activity at baseline was estimated using a previously validated 17-item questionnaire (Martínez-González et al., 2005). The level of physical activity was expressed in metabolic equivalents (METs), as detailed elsewhere (Basterra-Gortari et al., 2009).

### Dietary Interventions

Individualized energy requirements for each participant in the trial were estimated considering the resting energy expenditure and the physical activity level (Mifflin et al., 1990). Two hypocaloric diets (30% energy restriction) were prescribed during 16 weeks, which had different target macronutrient distribution based on previous trials (Handjieva-Darlenska et al., 2012; de la Iglesia et al., 2014). A low-fat (LF) diet provided 60% of total energy (E) from carbohydrates, 18% E from proteins, and 22% E from lipids following the NUGENOB trial criteria (Handjieva-Darlenska et al., 2012). On the other hand, a moderately high-protein (MHP) diet supplied 40% E from carbohydrates, 30% E from proteins, and 30% E from lipids according to the RESMENA trial criteria (de la Iglesia et al., 2014). No initial prescribed diets had less than 1,200 kcal/day. Both LF and MHP diets were designed on the basis of a food exchange system, where participants received detailed information from trained dietitians concerning portion sizes, feeding schedules, and cooking techniques, as described elsewhere (Goni et al., 2016).

Subjects were randomly assigned to one of the two diets through a specific algorithm designed for the study by MATLAB (http://www.mathworks.com) using stratifications according to sex, age groups (<45 and ≥45 years), and BMI (overweight, BMI 25–29.9 kg/m^2^; and obesity, BMI 30–40 kg/m^2^). Dietary adherence was evaluated based on expert dietitian’s criteria using the following scale: 3 = “very good adherence” (the participant strictly followed the diet at all times); 2 = “good adherence” (the participant occasionally exceeded from recommendations); 1 = “regular adherence” (the participant followed the diet across weekdays but not during weekend); and 0 = “poor adherence” (the participant does not follow the diet at any time).

In addition, the real macronutrient distribution of both MHP and LF diets was monitored using a 3-day weighed food record (including two weekdays and one weekday), which was applied at two times (8th and 16th weeks) during the period of the nutritional intervention. Moreover, dietitians conducted personal motivational telephone calls in order to increase the adherence to the dietary advice.

### Genotyping

Oral epithelium samples were collected with a liquid-based kit (ORAcollect-DNA, OCR-100, DNA Genotek Inc, Ottawa, Canada). Genomic DNA was isolated using the Maxwell^®^ 16 Buccal Swab LEV DNA Purification Kit (Promega Corp, Madison, WI, USA). A total of 95 SNPs related to weight loss, maintenance, and regains after multiple dietary prescriptions were selected through an exhaustive bibliographical review following the PRISMA criteria, as previously reported (Ramos-Lopez et al., 2018). The search included all available genome-wide association studies concerning obesity, weight loss, and energy metabolism. Genotyping was performed by targeted next-generation sequencing in the Ion Torrent PGM^™^ equipment (Thermo Fisher Scientific Inc, Waltham, MA, USA) using a pre-designed panel, as described elsewhere (Ramos-Lopez et al., 2018). The genomic characteristics of the 95 SNPs including minor allele frequencies and Hardy–Weinberg equilibrium have been recently reported (Ramos-Lopez et al., 2018).

### Statistical Analyses

Distribution of study variables (normality) was screened by the Kolmogorov–Smirnov test. Quantitative variables were expressed as means ± standard deviations, whereas categorical variables were presented as numbers and percentages. Principal variables including waist circumference reduction (WCR) and total body fat loss (TFATL) were normally distributed (*P* > 0.05). The sample size of the trial was estimated at 200 individuals (扴α = 0.05 and statistical power of 80%), assuming a mean difference in weight loss of 2 kg ± 3.5 kg. However, considering a potential dropout rate of 30%, it was considered necessary to recruit about 260 subjects. Statistical differences in anthropometric and biochemical changes after dietary intervention by sex and age were estimated using Student’s *t*-tests. Multiple linear regression models were used to predict WCR and TFATL changes in each dietary group through three accepted statistical approaches: least-angle regression (LARS) as previously described (Efron et al., 2004); best subset regression procedure (BSRP) as currently theorized (Lindsey and Sheather, 2010); and the bootstrapping stepwise method (BSM) as reported elsewhere (Austin and Tu, 2004).

The selection of SNPs to be incorporated into the models was performed according to the following steps. First, ANOVA tests were carried out to identify SNPs statistically or marginally associated with WCR and TFATL in each diet. Second, *post hoc* tests (Bonferroni’s and Dunnett’s T3) were run to define differences among genotypes in order to be differentially coded in “low risk = 0” and “high risk = 1” groups. A low-risk genotype was defined as the one that was related to higher values of WCR and TFATL (best response), whereas a high-risk genotype was that associated with lower values of WCR and TFATL (worst response). Genotypes with similar effects (*P* > 0.05) were clustered in a single category. In a third step, Student’s *t*-tests were further applied to confirm statistical differences between the categorized genotype groups (low risk vs. high risk), selecting those SNPs showing at least a marginal statistical trend (*P* < 0.10). SNPs with a low prevalence (<10%) in either genotype category (low risk vs. high risk) were excluded from the regression models.

To evaluate the combined effects of the previously selected SNPs on WCR and TFATL by MHP and LF dietary groups, four unweighted and four weighted genetic risk scores (GRSs) by main outcomes and diets (WCR-MHP diet; WCR-LF diet; TFATL-MHP diet; and TFATL-LF diet) were constructed through additive models, as described elsewhere (Hung et al., 2015). Briefly, the unweighted GRS (uGRS) was calculated by summation of the number of high-risk genotypes at each locus. Thus, each unit increase in the uGRS corresponded to one additional risk genotype. Instead, the weighted GRS (wGRS) was computed by multiplying the number of high-risk genotypes at each locus for the corresponding effect sizes (扴β-coefficients), in cm (WCR) or % (TFATL), and then summing the products. Both derived scores were based on the assumption that all SNPs of interest have independent effects and contribute in an additive manner on WCR and TFATL (Lin et al., 2009).

Both uGRS and wGRS were used as continuous variables in the multiple linear regression models. In addition to genetic variants, other conventional factors of personalization were evaluated including age, sex, and the following variables at baseline: physical activity (METs), energy intake (kcal), WC (cm), and TFAT (%). Eventual interactions of genes with environmental factors as well as with age and sex were evaluated by multivariate regression analyses.

Candidate models were tested to residual’s homoscedasticity (Ou et al., 2015). Also, multicollinearity between predictive variables was evaluated (Iacobucci et al., 2017). Furthermore, a correction for optimistic prediction and overfitting was performed according to Harrell’s bootstrapping algorithm (Harrell et al., 1996), which is based on using bootstrapped datasets to internally validate the multiple linear regression models as well as to repeatedly quantify the degree of overfitting in the model-building process. This method allows to select the best model showing the highest optimism-corrected adj. *R* ^2^ value. Moreover, squared partial correlations (PC^2^) were used to estimate the individual contribution of each predictor to the adiposity variability in response to diet. Statistical analyses were performed in the statistical program STATA 12 (StataCorp LLC, College Station, TX, USA; www.stata.com). Statistical significance was set at *P* < 0.05.

## Results

At the end of the trial, a total of 232 participants completed the nutritional intervention; however, those who presented poor dietary adherence (failure to follow the diet at any time) according to expert dietitian’s criteria (score = 0, see dietary interventions in the Materials and Methods section) were excluded (*n* = 31) from the statistical analyses (**Supplementary Figure 1**). Thus, 201 individuals (LF diet = 105 and MHP diet = 96) were further characterized at baseline and compared between MHP and LF diets (**Table 1**). The analysis of the macronutrient composition of the programmed diets revealed statistically significant differences in the distribution of proteins and fats (**Supplementary Table 1**).

**Table 1:** Anthropometric, biochemical, and dietary characteristics of the study participants at baseline according to assigned dietary groups.

Variable	MHP	LF	*P* value
*n*	96	105	–
Weight (kg)	86.8 ± 13.9	88.8 ± 12.2	0.288
BMI (kg/m^2^)	31.2 ± 3.1	32.2 ± 3.7	0.052
TFAT (%)	41.9 ± 5.5	41.9 ± 6.7	0.991
VFAT (kg)	1.39 ± 0.86	1.46 ± 0.81	0.551
WC (cm)	101.7 ± 10.7	102.8 ± 9.8	0.445
SBP (mmHg)	129.4 ± 19.6	128.1 ± 16.1	0.620
DBP (mmHg)	78.7 ± 11.1	80.0 ± 10.8	0.404
Glucose (mg/dL)	95.4 ± 9.7	95.8 ± 10.9	0.775
TC (mg/dL)	214.6 ± 37.4	217.8 ± 39.9	0.547
HDL-c (mg/dL)	54.1 ± 12.7	56.2 ± 13.3	0.252
TG (mg/dL)	100.7 ± 51.6	103.0 ± 57.9	0.768
TyG index	4.53 ± 0.24	4.54 ± 0.25	0.833
Energy (kcal/day)	2,933 ± 778	3,032 ± 1,029	0.447
Carbohydrate (%E/day)	40.2 ± 6.5	40.8 ± 6.6	0.528
Protein (%E/day)	16.7 ± 2.7	17.0 ± 3.0	0.471
Fat (%E/day)	41.2 ± 6.0	40.2 ± 5.7	0.239
Physical activity (METs)	23.7 ± 20.0	24.6 ± 18.2	0.740

Variables are expressed as means ± standard deviations. MHP, moderately high protein; LF, low fat; BMI, body mass index; TFAT, total body fat loss; VFAT, visceral fat; WCR, waist circumference reduction; SBP, systolic blood pressure; DBP, diastolic blood pressure; TC, total cholesterol; HDL-c, high-density lipoprotein cholesterol; TG, triglycerides; TyG index, triglyceride-glucose index.

According to the BMI classification of the World Health Organization, 35% of subjects were overweight (*n* = 70), whereas 65% (*n* = 131) presented grade I and grade II obesity. At baseline, no statistically significant differences in all anthropometric and biochemical variables between MHP and LF dietary groups were found. Instead, individuals randomized to the MHP regime had higher initial consumptions of protein and fat as well as a lower intake of carbohydrates than those assigned to the LF diet (**Table 1**).

Comparisons of responses between the baseline and the end of both dietary interventions for all the phenotypes are presented (**Supplementary Table 2**). After energy restriction, both MHP and LF diets induced statistically significant decreases in adiposity, body composition, blood pressure, and lipid profiles (**Supplementary Table 2**). The average changes in main outcomes (WCR and TFATL) and other variables by diet are also reported (**Table 2**). Of note, no significant differences between MHP and LF diets for WCR (−8.9 vs. −9.7 cm, respectively, *P* = 0.231) and TFATL (−4.4 vs. −4.3 cm, respectively, *P* = 0.852) as well as for the rest of evaluated markers were found.

**Table 2:** Anthropometric and biochemical outcomes in response to MHP and LF diets at the end of the nutritional intervention period.

Variable	MHP	LF	*P* value
*n*	96	105	–
*Main findings*			
Δ TFATL (%)	−4.4 ± 0.3	−4.3 ± 0.3	0.852
Δ WCR (cm)	−8.9 ± 0.5	−9.7 ± 0.5	0.231
*Other findings*			
Δ Weight (kg)	−8.5 ± 0.4	−9.1 ± 0.4	0.235
Δ BMI (kg/m^2^)	−3.1 ± 0.1	−3.3 ± 0.1	0.253
Δ VFAT (kg)	−0.5 ± 0.03	−0.5 ± 0.02	0.502
Δ SBP (mmHg)	−12.4 ± 1.2	−10.9 ± 1.1	0.370
Δ DBP (mmHg)	−4.2 ± 1.0	−4.5 ± 1.0	0.856
Δ Glucose (mg/dL)	−4.2 ± 0.8	−4.5 ± 0.8	0.768
Δ TC (mg/dL)	−18.2 ± 2.6	−22.7 ± 2.5	0.210
Δ HDL-c (mg/dL)	−3.0 ± 0.8	−5.1 ± 0.7	0.052
Δ TG (mg/dL)	−19.2 ± 3.7	−14.2 ± 3.6	0.331
Δ TyG index	−0.12 ± 0.19	−0.09 ± 0.18	0.231

Variables are expressed as means ± standard deviations. MHP, moderately high protein; LF, low fat; BMI, body mass index; TFATL, total body fat loss; VFAT, visceral fat; WCR, waist circumference reduction; SBP, systolic blood pressure; DBP, diastolic blood pressure; TC, total cholesterol; HDL-c, high-density lipoprotein cholesterol; TG, triglycerides; TyG index, triglyceride-glucose index.

**Figure 1:**
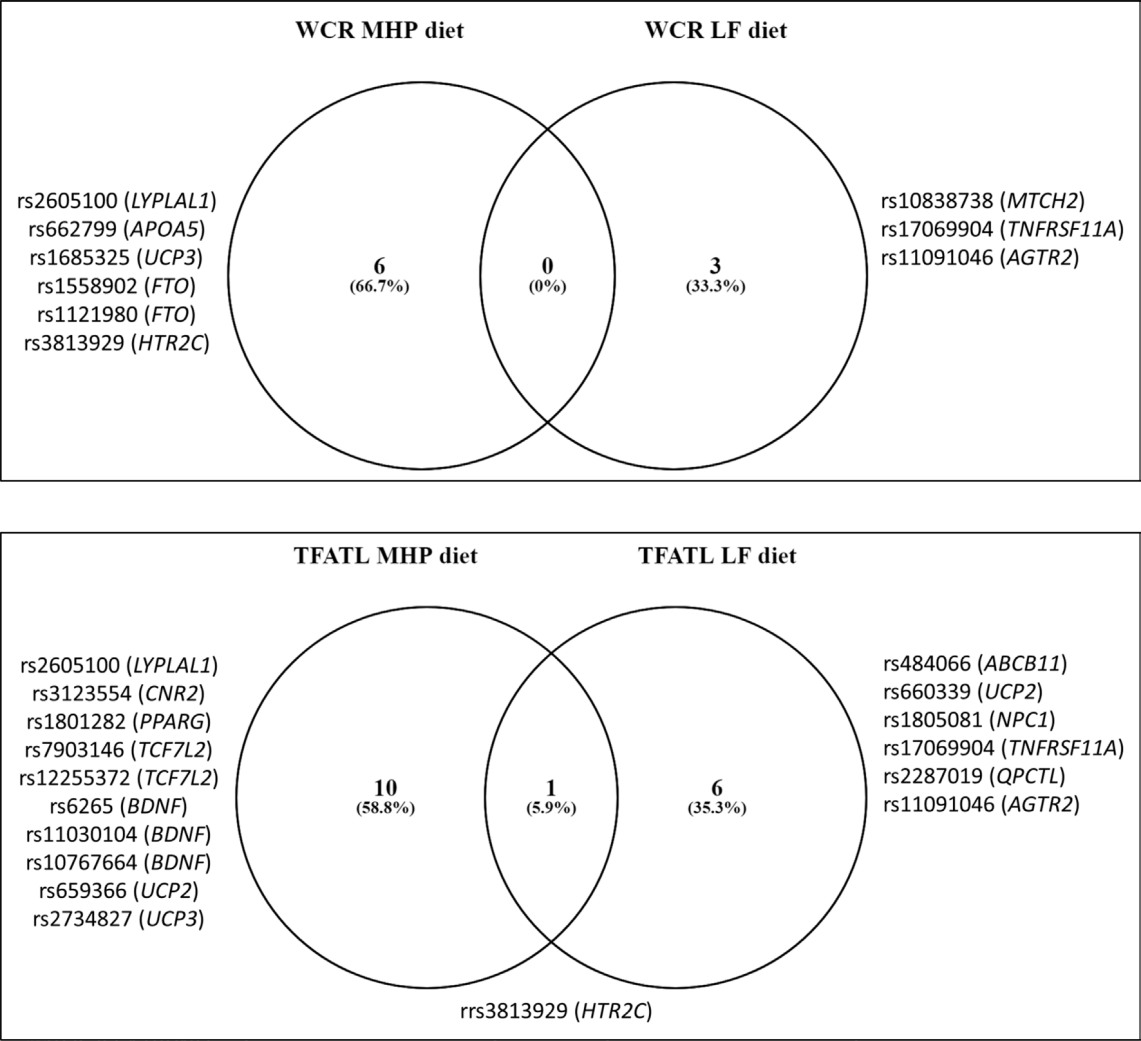
Venn diagram showing the number of SNPs associated with adiposity and body composition outcomes compared by diets. WCR, waist circumference reduction; TFATL, total body fat loss; MHP, moderately high protein; LF, low fat.

Mean values of the WCR and TFATL by genotypes of the 95 SNPs are reported (**Supplementary Tables 3a, b**, respectively). Overall, 26 different SNPs were associated with adiposity outcomes, 9 with WCR, and 17 with TFATL, most of which were specific for each dietary intervention (**Figure 1**). Six SNPs—rs2605100 (*LYPLAL1*), rs662799 (*APOA5*), rs1685325 (*UCP3*), rs1558902 (*FTO*), rs1121980 (*FTO*), and rs3813929 (*HTR2C*)—specifically accounted for differences in WCR within the MHP diet. On the other hand, in the LF diet, WCR diverged by genotypes of three genetic variants: rs10838738 (*MTCH2*), rs17069904 (*TNFRSF11A*), and rs11091046 (*AGTR2*). No common SNPs related to WCR variability between diets were found. In addition, 10 SNPs were particularly associated with TFATL in the MHP diet: rs2605100 (*LYPLAL1*), rs3123554 (*CNR2*), rs1801282 (*PPARG*), rs7903146 (*TCF7L2*), rs12255372 (*TCF7L2*), rs6265 (*BDNF*), rs11030104 (*BDNF*), rs10767664 (*BDNF*), rs659366 (*UCP2*), and rs2734827 (*UCP3*). In the LF diet, TFATL differed according to the following six SNPs: rs484066 (*ABCB11*), rs660339 (*UCP2*), rs1805081 (*NPC1*), rs17069904 (*TNFRSF11A*), rs2287019 (*QPCTL*), and rs11091046 (*AGTR2*). Only SNP rs3813929 (*HTR2C*) was commonly associated with TFATL in both diets.

The genotype codifications of the 26 relevant SNPs concerning WCR and TFATL changes according to dietary groups are presented (**Tables 3A**, B, respectively), which were further used to construct the corresponding four uGRS and four wGRS. Thus, the number of SNPs used for each GRS was as follows: WCR-MHP diet (*n* = 6), WCR-LF diet (*n* = 3), TFATL-MHP diet (*n* = 11), and TFAT-LF diet (*n* = 7), as reported in **Tables 3A**, B. The ranges of uGRS were WCR-MHP diet (0–6), WCR-LF diet (0–3), TFATL-MHP diet (0–11), and TFATL-LF diet (0–7). Regarding wGRS, the following ranges were calculated: WCR-MHP diet (−1.43 to 11.68), WCR-LF diet (0–6.83), TFATL-MHP diet (1.05–8.17), and TFATL-LF diet (0–7.51).

**Table 3A:** Genotype codifications of SNPs selected to construct genetic risk scores concerning WCR changes by dietary groups.

MHP diet	Genotypes		*n*		Means ± SD		*P* value
	Low risk	High risk	Low risk	High risk	Low risk	High risk	
rs2605100 (*LYPLAL1*)	GG	AA + AG	47	49	−9.8 ± 3.9	−8.0 ± 4.9	**0.046**
rs662799 (*APOA5*)	AA	GA	83	13	−9.2 ± 4.1	−6.8 ± 6.4	0.067
rs1685325 (*UCP3*)	TC + CC	TT	70	26	−9.4 ± 4.4	−7.5 ± 4.3	0.055
rs1558902 (*FTO*)	AA	TT + TA	16	80	−10.8 ± 4.9	−8.5 ± 4.3	0.067
rs1121980 (*FTO*)	GG + AA	GA	61	35	−9.7 ± 4.5	−7.4 ± 4.2	**0.014**
rs3813929 (*HTR2C*)	CC + TT	CT	80	16	−9.4 ± 4.5	−6.1 ± 3.2	**0.007**
**LF diet**							
rs10838738 (*MTCH2*)	AA	AG + GG	39	66	−10.7 ± 4.6	−8.8 ± 3.8	**0.028**
rs17069904 (*TNFRSF11A*)	GA + AA	GG	19	86	−11.6 ± 5.1	−9.1 ± 3.9	**0.017**
rs11091046 (*AGTR2*)	AA	AC + CC	27	78	−11.6 ± 4.8	−8.8 ± 3.8	**0.004**

Comparison of means between non-risk and risk genotypes by Student’s t-tests. Variables are expressed as number of cases or means ± SD. WCR, waist circumference reduction; MHP, moderately high protein; LF, low fat. Bold numbers indicate statistical significance.

**Table 3B:** Genotype codifications of SNPs selected to construct genetic risk scores concerning TFATL changes by dietary groups.

MHP diet	Genotypes		*n*		Means ± SD		*P* value
	Low risk	High risk	Low risk	High risk	Low risk	High risk	
rs2605100 (*LYPLAL1*)	GG	AA + AG	47	49	−4.9 ± 2.7	−3.8 ± 2.8	**0.050**
rs3123554 (*CNR2*)	AA + AG	GG	71	25	−4.7 ± 2.9	−3.5 ± 2.2	0.080
rs1801282 (*PPARG*)	CG	CC	12	84	−5.7 ± 3.7	−4.2 ± 2.6	0.075
rs7903146 (*TCF7L2*)	TT	CC + CT	16	80	−5.6 ± 2.4	−4.1 ± 2.8	**0.049**
rs12255372 (*TCF7L2*)	GT + TT	GG	65	31	−4.7 ± 2.9	−3.7 ± 2.5	0.089
rs6265 (*BDNF*)	CT + TT	CC	33	63	−5.3 ± 3.2	−3.9 ± 2.5	**0.023**
rs11030104 (*BDNF*)	AG + GG	AA	37	59	−5.2 ± 3.1	−3.8 ± 2.4	**0.013**
rs10767664 (*BDNF*)	TA	TT + AA	29	67	−5.7 ± 3.3	−3.8 ± 2.4	**0.002**
rs659366 (*UCP2*)	CC	CT + TT	48	48	−4.9 ± 3.0	−3.8 ± 2.5	0.071
rs2734827 (*UCP3*)	GG	GA + AA	46	50	−4.9 ± 3.0	−3.8 ± 2.5	0.056
rs3813929 (*HTR2C*)	CC + TT	CT	80	16	−4.6 ± 2.8	−3.1 ± 2.6	**0.041**
**LF diet**							
rs484066 (*ABCB11*)	TT	AA + AT	49	56	−4.8 ± 3.0	−3.9 ± 2.3	0.099
rs660339 (*UCP2*)	GA	GG + AA	54	51	−4.8 ± 2.7	−3.7 ± 2.5	**0.024**
rs1805081 (*NPC1*)	TT + TC	CC	87	18	−4.5 ± 2.8	−3.2 ± 1.6	0.095
rs17069904 (*TNFRSF11A*)	GA + AA	GG	19	86	−5.5 ± 3.5	−4.0 ± 2.4	0.053
rs2287019 (*QPCTL*)	CT	CC + TT	30	75	−4.8 ± 2.7	−4.1 ± 2.6	0.086
rs3813929 (*HTR2C*)	CC + TT	CT	87	18	−4.7 ± 2.7	−2.6 ± 1.7	**0.007**
rs11091046 (*AGTR2*)	AA + CC	AC	60	45	−5.0 ± 2.8	−3.4 ± 2.2	**0.003**

Comparison of means between non-risk and risk genotypes by Student’s t-tests. Variables are expressed as number of cases or means ± SD. TFATL, total body fat loss; MHP, moderately high protein; LF, low fat. Bold numbers indicate statistical significance.

WCR and TFATL predictions by diet were evaluated through multiple linear regression models using genetic (single SNPs, uGRS, and wGRS), phenotypic (age, sex, and WC or TFAT at baseline), and environment variables (physical activity level and energy intake at baseline) as well as potential interactions between genes and environmental factors. The statistical data of all candidate models are reported (**Supplementary Tables 4a–4d**), and the best models are summarized (**Table 4**). In general, higher optimism-corrected adj. *R* ^2^ values were obtained in models using uGRS or wGRS than in those incorporating single SNPs in addition to other environment and phenotypic variables. For example, the predictive values of models evaluating SNPs to explain WCR variance in the MHP diet ranked from 6 to 10%, which increased to 21% (optimism-corrected adj. *R* ^2^ = 0.2081) with wGRS, baseline WC, and age (**Supplementary Table 4a**). Similarly, single SNPs predicted 10–21% of TFATL variance in the MHP diet, whose performance improved to 32% (optimism-corrected adj. *R* ^2^ = 0.3208) when introducing wGRS, baseline TFAT, and age (**Supplementary Table 4b**). Also, uGRS and statistical interaction with physical activity as well as sex, physical activity, and energy intake at baseline were major contributors of WCR variance in the LF diet (about 22%, optimism-corrected adj. *R* ^2^ = 0.2208) compared with SNPs (13–17%, **Supplementary Table 4c**). Instead, SNPs accounted to only 22–29% of TFATL variance in the LF diet, whereas wGRS, TFAT, and energy intake at baseline, as well as interactions of wGRS with energy and TFAT, explained this trait in approximately 38% (optimism-corrected adj. *R* ^2^ = 0.3792, **Supplementary Table 4d**).

**Table 4:** Best multiple linear regression models using genetic, phenotypic, and environment information to explain WCR and TFATL outcomes as dependent variables by dietary groups.

Predictors	WCR				TFATL			
	MHP	PC^2^	LF	PC^2^	MHP	PC^2^	LF	PC^2^
Age (years)	−0.052	0.014	–	–	−0.043	0.037	–	–
Sex	–	–	1.636	0.031	–	–	–	–
Baseline WC (cm)	−0.067	0.025	–	–	–	–	–	–
Baseline TFAT (%)	–	–	–	–	0.125	0.082	0.194	0.060
Baseline physical activity (METs)	–	–	0.182	0.078	–	–	–	–
Baseline energy intake (100 kcal)	–	–	−0.083	0.052	–	–	−0.107	0.053
uGRS	–	–	3.250	0.174	–	–	–	–
wGRS	0.959	0.222	–	–	0.915	0.276	2.490	0.051
uGRS × baseline physical activity (METs)	–	–	−0.081	0.088	–	–	–	–
wGRS × baseline energy intake	–	–	–	–	–	–	0.020	0.029
wGRS × baseline TFAT	–	–	–	–	–	–	−0.052	0.048
Constant	−3.304	–	−15.425	–	−12.092	–	−12.130	–

Data are expressed as β values and squared partial correlations (PC^2^). Hyphens represent “not significant” or “not applicable” variables within each model. NS, not significant; NA, not applicable; METs, metabolic equivalents; uGRS, unweighted genetic risk score; wGRS, weighted genetic risk score; WCR, waist circumference reduction; TFATL, total body fat loss; MHP, moderately high protein; LF, low fat.

The individual contributions of each predictor into the models by adiposity outcome and diets are reported (**Table 4**). Interestingly, the calculated uGRS and wGRS were the greatest contributors of WCR-MHP diet, WCR-LF diet, and TFATL-MHP diet, with about 22% (PC^2^ = 0.222), 17% (PC^2^ = 0.174), and 28% (PC^2^ = 0.276), respectively. Meanwhile, TFAT and energy intake at baselines as well as the corresponding wGRS had a higher impact in TFATL-LF diet, with approximately 6% (PC^2^ = 0.060), 5.3% (PC^2^ = 0.053), and 5.1% (PC^2^ = 0.051), respectively.

**Figure 2:**
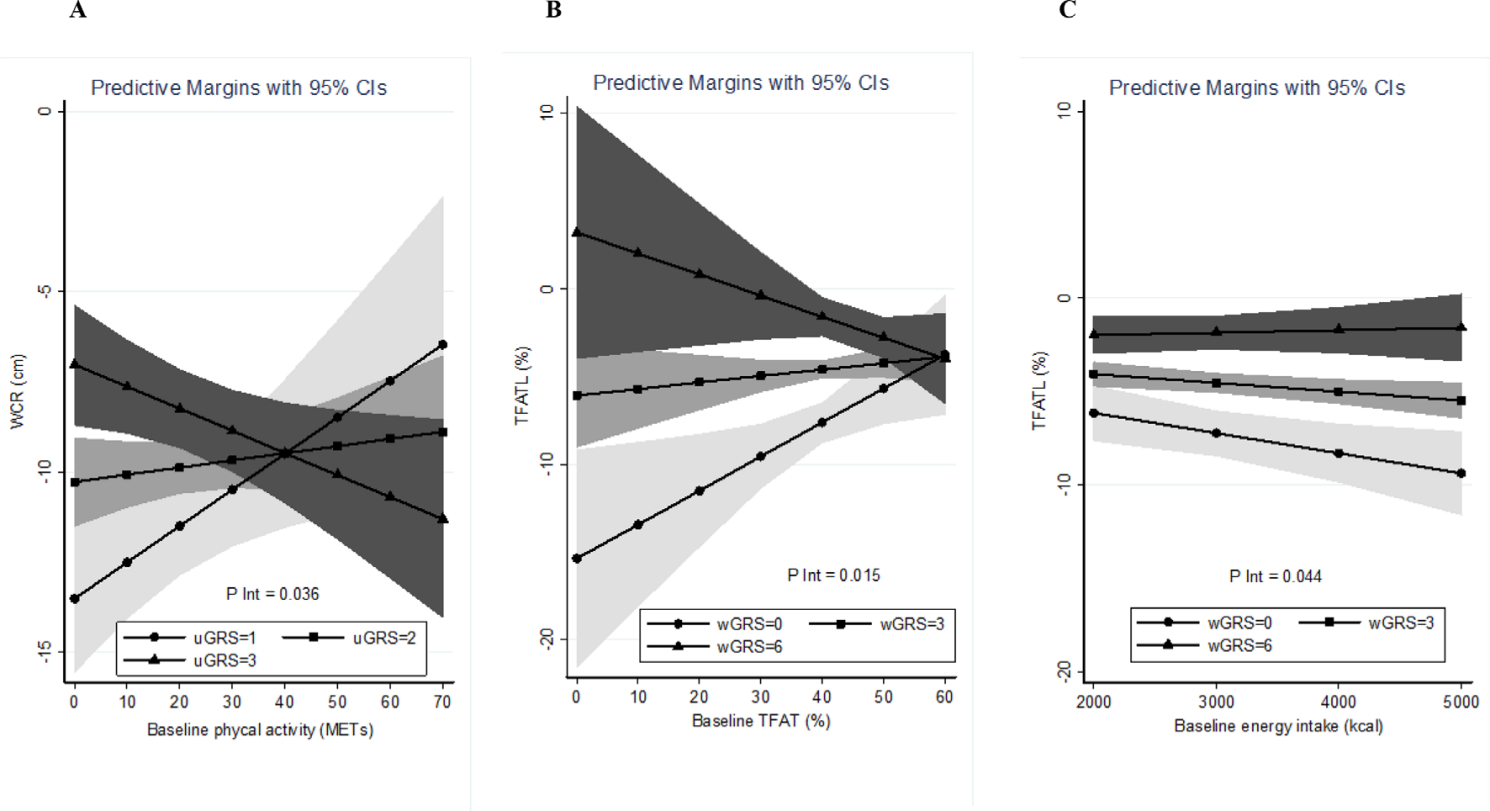
Estimations curves of gene–environment interactions concerning WCR and TFATL predictions in the LF diet. **(A)** Interaction between baseline physical activity and uGRS in relation to WCR. **(B)** Interaction between baseline TFAT and wGRS regarding TFATL. **(C)** Interaction between baseline energy intake and wGRS concerning TFATL. WCR, waist circumference reduction; TFATL, total body fat loss; uGRS, unweighted genetic risk score; wGRS, weighted genetic risk score.

Estimation curves of gene–environment interactions concerning WCR and TFATL predictions in the LF diet are plotted (**Figures 2A–C**). It is observed how the slopes of the studied variables (METs for WCR and baseline energy intake and baseline TFAT for TFATL) radically change depending on the value of the genetic score. Thus, in subjects carrying a high genetic risk (uGRS = 3), performing more physical activity (METs) was associated with greater WCR after the intervention (**Figure 2A**). Similarly, in participants who had a high genetic risk (wGRS = 6), greater baseline TFAT was related to higher TFATL (**Figure 2B**). Instead, in carriers of a low genetic risk (wGRS = 0), greater energy consumption was associated with higher TFATL (**Figure 2C**) at the end of the trial.

## Discussion

To date, different diets varying in macronutrient composition have been prescribed for weight control (Abete et al., 2010). In the current investigation, both MHP and LF diets induced statistically significant fat mass losses and blood marker reductions between baseline and after the interventions. Of note, no relevant differences in adiposity and metabolic outcomes between diets were found, which suggest that their effectiveness for the treatment of obesity was independent of the macronutrient content and was more driven by energy restriction.

Increasing evidence suggests that heterogeneity in individual responses to various energy-restricted diets in subjects with obesity is partially related to particular genetic profiles (Martínez and Milagro, 2015). In the present research, a total of 26 polymorphisms were independently associated with differential responses on WCR or TFATL according to the dietary prescriptions. Interestingly, most SNPs were specific for MHP or LF diets, which suggest that the genetic effect on adiposity outcomes is dependent on interactions with the macronutrient distribution. Only the rs3813929 (*HTR2C*) genetic variant had a similar effect on TFATL in both MHP and LF diets. Concerning WCR in the MHP diet, SNPs were located in genes related to lipid metabolism (*LYPLAL1* and *APOA5*); thermogenesis (*UCP3*); regulation of fat mass, adipogenesis, and energy homeostasis (*FTO*); and neurotransmitter release (*HTR2C*). Meanwhile, SNPs in genes implicated in mitochondrial membrane transport (*MTCH2*) and inflammation (*TNFRSF11A*) influenced WCR in the LF diet. Regarding TFATL in the MHP diet, SNPs were mapped to genes involved in lipid metabolism (*LYPLAL1* and *PPARG*), appetite (*CNR2*), Wnt signaling pathway (*TCF7L2*), neuronal cell differentiation (*BDNF*), thermogenesis (*UCP2* and *UCP3*), and neurotransmitter release (*HTR2C*). Instead, TFATL in the LF diet was modulated by SNPs in genes linked to bile secretion (*ABCB11*), thermogenesis (*UCP2*), cholesterol transport (*NPC1*), inflammation (*TNFRSF11A*), biosynthesis of pyroglutamyl peptides (*QPCTL*), angiotensin receptor (*AGTR2*), and neurotransmitter release (*HTR2C*). (https://www.genecards.org/).

Because the magnitude of associations between individual SNPs and metabolic traits is generally modest, multiple genetic testing based on the combination of several loci into a GRS is a common statistical method to correct the analytical value of single enetic variants (Janssens et al., 2006; Moonesinghe et al., 2010). In this trial, the predictive accuracy of all regression models substantially improved when combining all risk genotypes into a GRS instead of analyzing each single SNP separately. Accordingly, effect size estimations revealed that the calculated uGRS and wGRS were the greatest contributors (17–28%) to explain the variance in most phenotypes (WCR-MHP diet, WCR-LF diet, and TFATL-MHP diet), except for TFATL-LF diet, where TFAT and energy intake at baselines had a higher impact in this feature.

To date, few studies have evaluated the effect of GRS on diet-induced weight/body fat loss. According to our results, greater body fat loss was reported in carriers of an obesity GRS related to high sensitivity to changes in dietary habits or exercise in a large Korean cohort (Cha et al., 2018). Within the Look AHEAD (Action for Health in Diabetes) clinical trial, the highest GRS for diabetes susceptibility was associated with a greater WCR after 1 year of intensive lifestyle advice combining dietary fat restriction and increased physical activity, although the variance in change attributable to the GRS was small, ranging from 6.2% to 7.3% (Peter et al., 2012). Similarly, modest benefits in weight loss were found among participants with high GRS for coronary artery disease receiving standard of care based on diet plus exercise (Knowles et al., 2017). On the other hand, no relationships were found between a GRS constructed from genetic variants associating with BMI and changes in body weight during a 5-year follow-up intervention focused on changing habits of smoking, physical activity, dietary intake, and alcohol use in a Danish population (Sandholt et al., 2014). Also, diabetes GRS counseling did not significantly modify mean weight loss in overweight individuals following a 12-week group session program to improve diet quality and physical activity level (Grant et al., 2013).

Potential interactions between genetic and environmental factors also influence adiposity and body composition outcomes and need to be specifically considered (Qi, 2014). In this study, a statistically significant interaction between GRS and the level of physical activity at baseline was found concerning WCR only in the LF diet. Moreover, GRS interplayed with baseline energy intake and TFAT in relation to TFATL in this same dietary group. In a block-randomized clinical trial, significant interactions between resistance exercise and a GRS for obesity on weight and body fat reductions were found, where the putative effect of exercise in changes in body composition was greater among women with a lower GRS (Klimentidis et al., 2015). Conversely, body weight changes were influenced by dietary habits and physical activity over a 5-year follow-up lifestyle intervention, but independently of a GRS for high BMI (Sandholt et al., 2014). Given the little scientific evidence so far, further investigation is needed to analyze the role of multiple environment variables modulating the genetic contribution to individual dietary responses and to precision nutrition management (Ramos-Lopez et al., 2017).

Other relevant findings from this research were the involvement of some personalized phenotypic variables (age, sex, and the individual baseline anthropometric values) impacting WCR or TFATL outcomes. Thus, age influenced WCR and TFATL in the MHP diet, but not in the LF regime. Instead, exclusively, WCR in the LF diet was modulated by sex. Also, WC at baseline only contributed to explain WCR variance in the MHP diet, whereas baseline TFAT has an effect in TFATL in both dietary groups. The results suggest the need to consider age, sex, and body fat status to individualize dietary prescriptions in addition to the genetic background within tailored precision nutrition.

Similar results have been found in other clinical trials consisting of long-term lifestyle interventions based on hypocaloric LF diets and increased physical activity. For instance, findings from the Look AHEAD study (Look AHEAD Research Group, 2014), the Preventing Overweight Using Novel Dietary Strategies (POUNDS Lost) trial (Bray et al., 2017), and the Diabetes Prevention Program (DPP) approach (Delahanty et al., 2013) revealed that sex (men) and older age are generally associated with more successful end-of-study weight loss. In line with the results obtained in this research regarding TFATL, other trials such as the dietary intervention randomized controlled (DIRECT) trial (Greenberg et al., 2009) and the Sibutramine Trial of Obesity Reduction and Maintenance (STORM) study (Hansen et al., 2001) as well as a systematic review of clinical studies (Finkler et al., 2012) have also reported that higher initial body weight predicts greater rate of weight loss.

An important strength of this investigation was the construction of integrative models to cover genetic (multiple SNPs), phenotypic (age, sex, and initial anthropometric values), and environmental factors (energy intake and physical activity), as well as potential gene–environment interactions affecting diet-induced changes in two adiposity markers (WC and total fat mass). The contribution of all these factors to modulate adiposity outcomes under energy restriction ranging from 21% to up to 38% raises the need to be taken into account not only to predict the resistance/responsiveness to weight loss but also to personalize dietary recommendations for obesity management through a precision nutrition approach (Ramos-Lopez et al., 2017). In any case, this approach can be considered an early effort to personalize diets, which may change with scientific advances concerning gene–environment interactions. Additionally, because this study enrolled just European individuals, the possible role of population stratification in our results was minimized. Moreover, that fact that no differences between diets were found concerning anthropometric and metabolic markers at baseline suggests an adequate randomization process in this trial. On the other hand, the drawbacks of this research comprised the use of only two energy-restricted diets for weight loss, a relatively small sample analyzed, and a short time of follow-up. Thus, clinical trials including a larger number of subjects, additional diets with distinct macronutrient composition, and long times of follow-up are convenient. Furthermore, interactions of SNPs with other biological factors affecting body weight homeostasis such as microbiota composition, epigenetic phenomena, and metabolomic profiles need to be explored (Goni et al., 2016).

This investigation can be considered as a pioneer model study, where the interactions of genes and diet were analyzed in order to customize dietary prescriptions to produce more personalized benefits; however, scientific advances should complement the current instrument.

In conclusion, the interplay between different genetic variants and interactions with environmental factors modulate the individual responses to MHP and LF dietary interventions. These approaches are useful to optimize personalized nutritional strategies for the prevention and management of excessive adiposity through precision nutrition taking into account not only the genetic background but also lifestyle/clinical factors in addition to age and sex.

## Data Availability

The raw data supporting the conclusions of this manuscript will be made available by the authors, without undue reservation, to any qualified researcher.

## Ethics Statement

The study protocol was approved by the Research Ethics Committee of the University of Navarra (Ref. 132/2015). The research was performed in accordance with the ethical principles of the 2013 Helsinki Declaration. Participants voluntarily provided a written informed consent after they were informed about the details and procedures of the protocol.

## Author Contributions

JAM conceived the study, revised the data, and wrote and critically revised the content of this article. OR-L and JIR-B performed the statistical analysis and prepared the first draft of the manuscript. FM wrote and critically revised the content of this article. LG and MC recruited patients and performed the nutritional intervention. All authors critically reviewed all drafts and approved the final manuscript.

## Funding

This investigation was supported by grants from the Government of Navarra (PT024, OBEKIT project), CIBERobn (CB12/03/30002), and MINECO (AGL2013-45554-R). OR-L was supported by a 2-year postdoctoral grant from National Council of Science and Technology, Mexico (CONACyT, CVU 444175), in cooperation with the PhD Program in Molecular Biology in Medicine, University of Guadalajara, Mexico (CONACyT, PNPC 000091), and the University of Navarra, Spain (LE/97).

## Conflict of Interest Statement

The authors declare that the research was conducted in the absence of any commercial or financial relationships that could be construed as a potential conflict of interest.

## Abbreviations

BMI, body mass index; BSM, bootstrapping stepwise method; BSRP, best subset regression procedure; DBP, diastolic blood pressure; HDL-c, high-density lipoprotein cholesterol; LARS, least-angle regression; LDL-c, low-density lipoprotein cholesterol; LF, low fat; METs, metabolic equivalents; MHP, moderately high protein; SBP, systolic blood pressure; SNPs, single nucleotide polymorphisms; TC, total cholesterol; TFAT, total body fat; TFATL, total body fat loss; TG, triglycerides; TyG index, triglyceride-glucose index; uGRS, unweighted genetic risk score; VFAT, visceral fat; WC, waist circumference; WCR, waist circumference reduction; wGRS, weighted genetic risk score.
