# Supplementary material for: Models Integrating Genetic and Lifestyle Interactions on Two Adiposity Phenotypes for Personalized Prescription of Energy-Restricted Diets With Different Macronutrient Distribution
Source: Front Genet. 2019 Jul 30;10:686. doi: 10.3389/fgene.2019.00686 (PMC6683656; doi:10.3389/fgene.2019.00686)
Supplement: Supplementary file 1 [file DataSheet_1.docx]

**Supplementary Table 1.** Comparisons of real macronutrient distributions of MHP and LF diets at the end of both nutritional interventions.

| Variable | MHP | LF | *P* value |
| --- | --- | --- | --- |
| n= | 96 | 105 | - |
| Energy (Kcal/d) | 1340±274 | 1327±238 | 0.720 |
| Carbohydrate (%E/d) | 42.1±4.4 | 54.5±7.1 | **<0.001** |
| Protein (%E/d) | 28.4±3.9 | 21.4±3.3 | **<0.001** |
| Fat (%E/d) | 31.5±4.5 | 26.4±5.6 | **<0.001** |

Variables are expressed as means ± standard deviations. MHP: moderately high-protein; LF: low-fat. Bold numbers indicate statistically significant.

**Supplementary Table 2**. Anthropometric and biochemical characteristics of the study participants by diet at baseline and at the end of the intervention period.

| Variable | MHP | | | LF | | |
| --- | --- | --- | --- | --- | --- | --- |
|  | Initial | Final | *P* value | Initial | Final | *P* value |
| Weight (kg) | 86.8±13.9 | 78.4±13.6 | **<0.001** | 88.8±12.2 | 79.7±11.4 | **<0.001** |
| BMI (kg/m^2^) | 31.2±3.1 | 28.2±3.2 | **<0.001** | 32.2±3.7 | 28.9±3.6 | **<0.001** |
| TFAT (%) | 41.9±5.5 | 37.6±6.9 | **<0.001** | 41.9±6.7 | 37.6±8.1 | **<0.001** |
| VFAT (kg) | 1.39±0.86 | 0.93±0.61 | **<0.001** | 1.46±0.81 | 0.98±0.58 | **<0.001** |
| WC (cm) | 101.7±10.7 | 93.2±10.8 | **<0.001** | 102.8±9.8 | 92.9±10.4 | **<0.001** |
| SBP (mmHg) | 129.4±19.6 | 116.5±14.7 | **<0.001** | 128.1±16.1 | 117.6±12.9 | **<0.001** |
| DBP (mmHg) | 78.7±11.1 | 74.2±9.0 | **<0.001** | 80.0±10.8 | 75.3±7.6 | **<0.001** |
| Glucose (mg/dL) | 95.4±9.7 | 91.2±8.3 | **<0.001** | 95.8±10.9 | 91.1±8.6 | **<0.001** |
| TC (mg/dL) | 214.6±37.4 | 196.4±36.6 | **<0.001** | 217.8±39.9 | 195.1±39.6 | **<0.001** |
| HDL-c (mg/dL) | 54.1±12.7 | 51.1±11.0 | **<0.001** | 56.2±13.3 | 51.2±10.5 | **<0.001** |
| TG (mg/dL) | 100.7±51.6 | 81.7±44.5 | **<0.001** | 103.0±57.9 | 88.8±49.4 | **<0.001** |
| TyG index | 4.53±0.24 | 4.41±0.22 | **<0.001** | 4.54±0.25 | 4.45±0.23 | **<0.001** |

Variables are expressed as means ± standard deviations. MHP: moderately high-protein; LF: low-fat; BMI: body mass index; TFAT: total body fat loss; VFAT: visceral fat; WC: waist circumference reduction; SBP: systolic blood pressure; DBP: diastolic blood pressure; TC: total cholesterol; HDL-c: high-density lipoprotein cholesterol; TG: triglycerides; TyG index: triglyceride-glucose index. Bold numbers indicate statistically significant.

**Supplementary Table 3a.** Waist circumference reduction (WCR) according to genotypes of the 95 single SNPs by diet.

|  | MHP diet | | |  | LF diet | | |  |
| --- | --- | --- | --- | --- | --- | --- | --- | --- |
| SNP | 0 | 1 | 2 | *P* | 0 | 1 | 2 | *P* |
| rs17024393 (*GNAT2*) | -8.6±5.0 | -13.1±6.9 | -17.7 | 0.098 | -9.7±4.3 | -8.6±1.2 | - | 0.657 |
| rs1801131 (*MTHFR*) | -9.5±4.8 | -8.5±3.8 | -5.3±11.8 | 0.185 | -10.0±4.4 | -9.3±4.4 | -10.1±1.5 | 0.736 |
| rs1801133 (*MTHFR*) | -8.3±5.8 | -8.7±4.3 | -10.5±5.7 | 0.392 | -10.3±4.5 | -9.3±4.1 | -9.7±4.4 | 0.583 |
| rs543874 (*SEC16B*) | -9.2±4.4 | -7.1±6.8 | -11.6±6.0 | 0.186 | -9.4±4.1 | -10.1±4.7 | - | 0.513 |
| rs2605100 (*LYPLAL1*) | -9.8±4.1 | -7.6±6.3 | -9.6±3.9 | 0.191 | -6.7±4.1 | -10.0±4.4 | -9.7±4.2 | 0.350 |
| rs4846567 (*LYPLAL1*) | -9.2±4.3 | -8.4±6.1 | -8.7±3.2 | 0.782 | -9.6±3.8 | -9.2±4.8 | -11.8±1.6 | 0.406 |
| rs3123554 (*CNR2*) | -9.6±4.8 | -9.2±4.5 | -7.6±6.1 | 0.320 | -9.2±4.1 | -10.4±4.6 | -8.8±3.6 | 0.233 |
| rs324420 (*FAAH*) | -8.9±5.4 | -8.7±4.5 | -8.1±4.2 | 0.957 | -9.7±4.9 | -9.5±3.3 | -10.1±2.3 | 0.948 |
| rs8179183 (*LEPR*) | -9.1±4.3 | -8.2±7.1 | -7.2 | 0.735 | -9.9±4.3 | -8.7±4.0 | -14.0±0.1 | 0.173 |
| rs2815752 (*NEGR1*) | -10.4±3.7 | -8.6±5.8 | -8.9±4.4 | 0.666 | -10.0±4.8 | -9.7±4.8 | -9.5±3.4 | 0.947 |
| rs519887 (*ABCB11*) | -9.0±4.3 | -8.8±4.3 | -8.7±8.1 | 0.982 | -9.0±3.9 | -9.5±3.8 | -10.7±5.4 | 0.340 |
| rs484066 (*ABCB11*) | -9.0±3.5 | -9.3±4.5 | -8.0±6.5 | 0.578 | -8.7±3.4 | -9.1±3.7 | -10.5±4.9 | 0.222 |
| rs569805 (*ABCB11*) | -8.9±3.7 | -9.4±4.6 | -8.2±6.1 | 0.614 | -8.9±3.7 | -8.8±3.6 | -10.5±4.7 | 0.156 |
| rs494874 (*ABCB11*) | -9.5±3.8 | -9.4±4.4 | -8.0±6.1 | 0.465 | -8.9±3.7 | -8.8±3.7 | -10.5±4.7 | 0.162 |
| rs2943641 (*IRS1*) | -8.2±4.4 | -9.5±4.8 | -8.4±5.4 | 0.572 | -8.5±2.8 | -9.7±4.9 | -10.1±3.7 | 0.540 |
| rs10182181 (*ADCY3*) | -8.7±8.5 | -8.7±4.0 | -9.3±3.6 | 0.899 | -8.4±3.6 | -10.0±4.8 | -10.3±3.4 | 0.288 |
| rs713586 (*ADCY3*) | -9.1±8.5 | -8.6±4.0 | -9.3±3.6 | 0.834 | -8.4±3.6 | -9.9±4.8 | -10.4±3.4 | 0.265 |
| rs2860323 (*TMEM18*) | -9.6±6.9 | -9.7±4.9 | -8.4±5.0 | 0.577 | -6.9 | -10.3±5.1 | -9.5±3.9 | 0.553 |
| rs2867125 (*TMEM18*) | -9.6±6.9 | -9.7±4.9 | -8.7±3.9 | 0.666 | -6.9 | -10.4±5.2 | -9.6±3.7 | 0.564 |
| rs13021737 (*TMEM18*) | -9.6±6.9 | -9.5±4.9 | -8.5±5.0 | 0.687 | -6.9 | -10.5±5.2 | -9.4±3.8 | 0.403 |
| rs1801282 (*PPARG*) | -8.6±5.0 | -10.4±5.2 | - | 0.294 | -9.7±4.4 | -9.9±4.0 | -8.4 | 0.937 |
| rs2959272 (*PPARG*) | -7.5±6.1 | -9.6±4.7 | -9.1±4.0 | 0.220 | -8.9±4.2 | -9.8±4.3 | -10.3±4.3 | 0.470 |
| rs1386835 (*PPARG*) | -9.2±3.6 | -8.1±5.9 | - | 0.339 | -10.1±4.1 | -9.4±4.7 | - | 0.450 |
| rs709158 (*PPARG*) | -9.6±4.0 | -8.4±6.0 | -8.3±4.6 | 0.566 | -10.1±4.0 | -9.6±4.8 | -7.8±1.6 | 0.304 |
| rs1175540 (*PPARG*) | -9.7±4.0 | -8.4±6.0 | -8.3±4.6 | 0.479 | -10.3±4.1 | -9.6±4.7 | -7.8±1.6 | 0.274 |
| rs1175544 (*PPARG*) | -9.3±3.9 | -8.6±6.1 | -8.3±4.6 | 0.766 | -10.3±4.0 | -9.5±4.9 | -8.1±2.0 | 0.299 |
| rs1797912 (*PPARG*) | -9.4±4.2 | -8.5±5.7 | -8.7±4.4 | 0.748 | -10.0±3.9 | -9.7±5.1 | -8.7±2.5 | 0.604 |
| rs1516725 (*ETV5*) | -6.5±4.2 | -8.5±4.7 | -9.0±5.2 | 0.761 | -11.4 | -9.1±4.6 | -9.8±4.2 | 0.771 |
| rs9816226 (*ETV5*) | -6.7±3.6 | -9.1±4.9 | -9.0±5.2 | 0.607 | -11.4 | -9.0±4.4 | -10.0±4.2 | 0.544 |
| rs13107325 (*SLC39A8*) | -9.2±5.1 | -6.0±3.3 | - | 0.070 | -9.6±4.3 | -9.9±4.1 | - | 0.789 |
| rs1799883 (*FABP2*) | -8.5±4.7 | -8.7±6.1 | -9.0±4.5 | 0.935 | -9.1±6.6 | -9.9±4.7 | -9.6±3.7 | 0.912 |
| rs6536991 (*UCP1*) | -9.3±4.3 | -7.6±6.2 | -11.6±5.0 | 0.212 | -9.7±4.2 | -9.7±4.4 | -9.5±4.7 | 0.995 |
| rs12502572 (*UCP1*) | -9.0±4.3 | -8.5±5.9 | -9.5±6.3 | 0.867 | -9.7±4.3 | -9.5±4.4 | -9.9±4.3 | 0.967 |
| rs1800592 (*UCP1*) | -9.3±4.3 | -7.9±6.5 | -10.4 | 0.457 | -9.3±4.3 | -10.6±4.4 | -10.4±5.7 | 0.367 |
| rs8192678 (*PPARGC1A*) | -9.6±4.1 | -8.6±4.6 | -6.9±8.5 | 0.293 | -9.3±3.6 | -10.1±4.6 | -10.1±5.4 | 0.655 |
| rs10938397 (*GNPDA2*) | -6.3±7.7 | -8.9±4.3 | -9.0±4.4 | 0.262 | -10.8±5.3 | -9.4±4.1 | -9.5±4.4 | 0.508 |
| rs1801260 (*CLOCK*) | -8.2±5.8 | -9.4±4.3 | -10.6±3.3 | 0.395 | -9.7±4.1 | -9.9±5.1 | -8.8±2.5 | 0.773 |
| rs1440581 (*PPM1K*) | -8.0±6.3 | -9.6±4.7 | -8.4±4.4 | 0.421 | -9.6±5.7 | -10.2±3.6 | -9.0±4.4 | 0.449 |
| rs1042713 (*ADRB2*) | -8.8±4.2 | -9.3±5.9 | -6.7±3.3 | 0.460 | -9.2±4.9 | -10.1±3.9 | -8.9±4.0 | 0.570 |
| rs1042714 (*ADRB2*) | -9.9±4.7 | -8.6±5.2 | -8.1±2.2 | 0.638 | -9.6±4.0 | -9.7±4.1 | -9.6±6.8 | 0.999 |
| rs6861681 (*CPEB4*) | -9.0±4.4 | -9.6±4.6 | -5.6±8.4 | 0.110 | -10.2±4.9 | -9.2±3.5 | -9.2±4.2 | 0.549 |
| rs1800629 (*TNFA*) | -8.9±5.0 | -8.2±4.5 | -21.0 | **0.046** | -9.5±4.2 | -10.5±4.4 | -6.5 | 0.475 |
| rs206936 (*NUDT3*) | -8.4±5.2 | -9.7±4.8 | -9.1±5.0 | 0.560 | -10.0±4.6 | -9.1±3.9 | -10.5±3.7 | 0.542 |
| rs987237 (*TFAP2B*) | -8.5±5.4 | -9.9±4.4 | -6.1±1.4 | 0.383 | -9.2±4.1 | -10.8±4.6 | -13.7±4.4 | 0.119 |
| rs2207139 (*TFAP2B*) | -8.5±5.3 | -10.0±4.5 | -6.1±1.4 | 0.329 | -9.2±4.1 | -11.0±4.8 | -10.6 | 0.205 |
| rs7799039 (*LEP*) | -8.6±4.5 | -9.3±4.3 | -8.3±6.4 | 0.747 | -9.7±4.7 | -9.5±4.4 | -9.8±3.6 | 0.966 |
| rs4731426 (*LEP*) | -9.3±5.4 | -9.5±4.1 | -8.2±5.7 | 0.507 | -11.0±4.3 | -9.3±4.8 | -9.6±3.5 | 0.467 |
| rs2071045 (*LEP*) | -9.0±5.8 | -9.3±4.0 | -6.1±1.6 | 0.448 | -10.1±4.6 | -9.4±4.0 | -7.7±2.4 | 0.382 |
| rs1055144 (*NFE2L3*) | -8.9±5.5 | -8.7±4.1 | -12.4 | 0.771 | -9.9±4.6 | -8.9±3.3 | -13.7±1.6 | 0.234 |
| rs4994 (*ADRB3*) | -8.8±5.4 | -9.0±3.6 | - | 0.903 | -9.5±4.3 | -10.6±4.1 | - | 0.409 |
| rs1800544 (*ADRA2A*) | -10.9±4.1 | -8.5±5.9 | -8.7±4.4 | 0.445 | -13.8±6.5 | -9.9±3.4 | -8.7±4.0 | **0.003** |
| rs2419621 (*ACSL5*) | -9.6±4.6 | -8.2±5.8 | -6.1±3.6 | 0.267 | -9.4±4.3 | -10.0±4.4 | -9.4±2.4 | 0.756 |
| rs7903146 (*TCF7L2*) | -8.8±4.4 | -8.6±5.7 | -9.6±4.4 | 0.798 | -10.3±4.2 | -9.1±4.6 | -9.5±3.3 | 0.437 |
| rs12255372 (*TCF7L2*) | -8.8±4.4 | -8.8±5.8 | -9.2±4.3 | 0.956 | -10.1±4.3 | -9.3±4.5 | -9.2±3.3 | 0.667 |
| rs1800497 (*ANKK1*) | -9.0±4.3 | -8.6±6.5 | -8.6±3.1 | 0.920 | -9.3±4.1 | -10.5±4.6 | -11.0±2.7 | 0.398 |
| rs662799 (*APOA5*) | - | -7.5±6.0 | -9.1±4.9 | 0.350 | -10.8 | -9.4±4.3 | -9.7±4.3 | 0.937 |
| rs6265 (*BDNF*) | -8.9±4.3 | -8.6±6.6 | -9.4±4.5 | 0.923 | -9.3±3.9 | -10.3±5.1 | -10.2±3.1 | 0.586 |
| rs11030104 (*BDNF*) | -9.0±4.3 | -8.7±6.7 | -8.5±4.0 | 0.954 | -9.4±3.9 | -10.3±4.9 | -8.9±4.0 | 0.572 |
| rs10767664 (*BDNF*) | -8.6±4.9 | -9.2±7.1 | -8.6±3.6 | 0.883 | -8.4±3.4 | -10.6±4.8 | -9.2±4.0 | 0.252 |
| rs11605924 (*CRY2*) | -8.4±3.7 | -8.8±5.6 | -9.6±5.3 | 0.797 | -8.7±3.8 | -10.4±4.5 | -9.4±4.2 | 0.288 |
| rs10838738 (*MTCH2*) | -8.6±5.9 | -9.1±4.7 | -9.1±2.9 | 0.926 | -10.9±4.7 | -9.2±3.8 | -8.5±4.1 | 0.096 |
| rs660339 (*UCP2*) | -8.5±5.8 | -9.4±4.6 | -8.2±4.0 | 0.667 | -9.3±4.2 | -10.3±4.6 | -8.4±2.9 | 0.260 |
| rs659366 (*UCP2*) | -8.6±5.7 | -9.6±4.4 | -7.7±3.9 | 0.516 | -9.4±4.0 | -10.3±4.7 | -8.4±3.0 | 0.325 |
| rs2075577 (*UCP3*) | -6.9±6.9 | -9.3±4.1 | -9.7±4.9 | 0.169 | -10.1±3.6 | -9.4±4.0 | -9.9±5.1 | 0.777 |
| rs2734827 (*UCP3*) | -9.3±4.6 | -8.3±5.7 | -8.9±4.6 | 0.690 | -9.9±3.6 | -9.8±4.9 | -8.4±4.1 | 0.513 |
| rs1685325 (*UCP3*) | -6.5±6.4 | -10.0±4.2 | -9.0±4.5 | **0.027** | -9.7±3.4 | -10.0±4.0 | -9.0±5.5 | 0.690 |
| rs2075576 (*UCP3*) | -8.5±5.3 | -9.9±4.5 | -6.4±3.9 | 0.289 | -9.8±4.0 | -9.7±5.1 | -7.6±1.1 | 0.692 |
| rs1800006 (*UCP3*) | -8.4±5.3 | -10.1±4.5 | -6.4±3.9 | 0.218 | -9.8±4.0 | -9.7±5.3 | -8.2±2.2 | 0.736 |
| rs1800849 (*UCP3*) | -8.5±5.3 | -9.9±4.5 | -6.4±3.9 | 0.289 | -9.6±4.2 | -10.2±4.7 | -7.6±1.1 | 0.605 |
| rs4929949 (*STK33*) | -8.3±7.0 | -9.5±4.4 | -8.2±4.1 | 0.547 | -10.1±5.0 | -9.6±3.8 | -9.5±4.4 | 0.846 |
| rs10830963 (*MTNR1B*) | -8.9±5.6 | -8.1±4.2 | -12.3±3.7 | 0.189 | -9.6±4.0 | -10.0±4.7 | -8.4±4.0 | 0.698 |
| rs4769873 (*ALOX5AP*) | -8.5±5.3 | -9.9±4.2 | -11.7±6.4 | 0.414 | -10.0±4.3 | -8.3±4.0 | -10.2 | 0.349 |
| rs1052700 (*PLIN1*) | -8.5±4.3 | -9.1±4.4 | -9.3±8.6 | 0.847 | -9.3±4.5 | -10.0±4.1 | -9.8±3.9 | 0.755 |
| rs894160 (*PLIN1*) | -8.8±5.6 | -9.1±4.5 | -7.6±4.5 | 0.784 | -9.6±4.3 | -10.3±4.2 | -6.3±2.9 | 0.138 |
| rs2289487 (*PLIN1*) | -9.2±4.9 | -9.3±4.2 | -8.0±6.0 | 0.549 | -8.7±3.7 | -10.0±4.2 | -9.6±4.5 | 0.731 |
| rs7498665 (*SH2B1*) | -8.4±5.5 | -9.6±4.3 | -10.9±3.1 | 0.396 | -10.0±4.6 | -9.9±4.1 | -8.2±3.8 | 0.368 |
| rs7359397 (*SH2B1*) | -8.3±5.5 | -9.6±4.3 | -11.6±3.2 | 0.306 | -9.8±4.6 | -10.1±4.0 | -8.2±3.8 | 0.350 |
| rs1558902 (*FTO*) | -8.7±6.1 | -8.2±3.5 | -10.7±5.0 | 0.295 | -10.5±4.8 | -9.1±3.8 | -9.8±4.4 | 0.381 |
| rs1121980 (*FTO*) | -8.9±6.1 | -7.9±3.7 | -10.7±4.7 | 0.192 | -10.6±4.8 | -9.3±3.9 | -9.4±4.4 | 0.430 |
| rs17817449 (*FTO*) | -8.7±6.1 | -8.5±3.9 | -10.1±4.8 | 0.592 | -10.6±4.7 | -9.2±4.1 | -9.6±3.7 | 0.371 |
| rs8050136 (*FTO*) | -8.7±6.1 | -8.5±3.9 | -10.1±4.8 | 0.592 | -10.6±4.7 | -9.2±4.1 | -9.6±3.7 | 0.371 |
| rs3751812 (*FTO*) | -8.7±6.1 | -8.6±3.8 | -10.1±5.0 | 0.630 | -10.5±4.6 | -9.2±4.2 | -9.6±3.7 | 0.401 |
| rs9939609 (*FTO*) | -8.7±6.1 | -8.6±3.9 | -9.9±4.7 | 0.670 | -10.6±4.7 | -9.2±4.1 | -9.6±3.7 | 0.371 |
| rs12452844 (*AANAT*) | -10.0±4.3 | -8.1±5.7 | -7.1±2.9 | 0.184 | -9.4±4.2 | -10.2±4.4 | -8.8±4.5 | 0.628 |
| rs1805081 (*NPC1*) | -9.3±4.2 | -7.9±5.7 | -10.4±6.3 | 0.324 | -9.6±4.1 | -10.0±4.7 | -9.0±3.3 | 0.752 |
| rs6567160 (*MC4R*) | -9.4±4.3 | -7.2±6.7 | -10.9±1.1 | 0.173 | -9.4±4.5 | -9.8±3.9 | -11.5±3.8 | 0.494 |
| rs571312 (*MC4R*) | -9.4±4.3 | -7.2±6.7 | -10.9±1.1 | 0.173 | -9.4±4.5 | -9.9±3.9 | -11.5±3.8 | 0.487 |
| rs17782313 (*MC4R*) | -8.8±5.2 | -11.3±1.0 | -9.6 | 0.699 | -9.5±4.3 | -14.6±4.0 | -10.4±2.9 | 0.117 |
| rs17066866 (*MC4R*) | -8.8±5.2 | -9.4±1.2 | - | 0.843 | -9.7±4.3 | -7.9±1.4 | - | 0.553 |
| rs17069904 (*TNFRSF11A*) | -9.0±5.3 | -8.2±4.2 | - | 0.518 | -9.1±3.9 | -12.0±5.1 | -12.5 | **0.033** |
| rs2287019 (*QPCTL*) | -8.9±5.3 | -8.7±4.7 | - | 0.869 | -9.5±4.3 | -10.1±4.4 | - | 0.527 |
| rs6013029 (*CTNNBL1*) | -8.7±5.3 | -9.8±3.7 | - | 0.470 | -9.6±4.3 | -10.8±3.5 | -1.8 | 0.103 |
| rs6123837 (*GNAS*) | -9.9±5.2 | -8.1±5.3 | -9.2±3.4 | 0.343 | -8.7±4.4 | -10.2±3.9 | -10.2±4.9 | 0.286 |
| rs3813929 (*HTR2C*) | -9.0±5.3 | -6.7±3.1 | -11.6±4.1 | 0.081 | -9.8±4.5 | -9.2±4.1 | -10.0±3.0 | 0.846 |
| rs11091046 (*AGTR2*) | -8.8±5.0 | -8.9±4.7 | -8.6±6.1 | 0.970 | -11.4±5.1 | -8.4±3.2 | -9.5±4.4 | **0.039** |

Comparison of means between genotypes by ANOVA tests. Variables are expressed as means ± SD. MHP: moderately high-protein; LF: low-fat. Bold numbers indicate statistically significant. 0 = wild-type homozygotes; 1 = heterozygotes; 2 = mutant homozygotes.

**Supplementary Table 3b.** Total body fat loss (TFATL) according to genotypes of the 95 single SNPs by diet.

|  | MHP diet | | |  | LF diet | | |  |
| --- | --- | --- | --- | --- | --- | --- | --- | --- |
| SNP | 0 | 1 | 2 | *P* | 0 | 1 | 2 | *P* |
| rs17024393 (*GNAT2*) | -4.3±2.7 | -7.6±4.6 | -4.5 | 0.124 | -4.3±2.6 | -3.8±0.14 | - | 0.789 |
| rs1801131 (*MTHFR*) | -4.4±2.9 | -3.9±2.3 | -6.1±3.5 | 0.168 | -4.3±2.6 | -4.3±2.7 | -3.6±2.4 | 0.794 |
| rs1801133 (*MTHFR*) | -4.4±2.7 | -4.1±2.8 | -5.1±3.0 | 0.484 | -4.4±2.9 | -4.1±2.4 | -4.7±2.5 | 0.797 |
| rs543874 (*SEC16B*) | -4.4±2.9 | -4.3±2.4 | -5.1±2.0 | 0.902 | -4.2±2.3 | -4.4±3.1 | -4.0±0.21 | 0.886 |
| rs2605100 (*LYPLAL1*) | -3.8±3.0 | -3.8±2.8 | -4.9±2.7 | 0.148 | -2.7±1.8 | -4.4±2.8 | -4.3±2.5 | 0.349 |
| rs4846567 (*LYPLAL1*) | -4.6±2.9 | -4.4±2.8 | -3.7±2.5 | 0.690 | -4.4±2.5 | -4.3±2.7 | -4.4±3.0 | 0.984 |
| rs3123554 (*CNR2*) | -5.2±3.3 | -4.4±2.7 | -3.5±2.2 | 0.108 | -4.4±2.6 | -4.2±2.7 | -4.3±2.4 | 0.934 |
| rs324420 (*FAAH*) | -4.4±2.7 | -4.3±3.1 | -3.6±2.7 | 0.924 | -4.3±2.8 | -4.2±2.1 | -4.7±3.6 | 0.917 |
| rs8179183 (*LEPR*) | -4.4±2.9 | -4.3±2.5 | -1.3 | 0.549 | -4.5±2.8 | -3.7±2.1 | -4.8±3.5 | 0.427 |
| rs2815752 (*NEGR1*) | -3.2±2.1 | -4.5±2.9 | -4.4±2.8 | 0.539 | -4.6±2.7 | -4.1±2.8 | -4.4±2.2 | 0.702 |
| rs519887 (*ABCB11*) | -4.6±3.2 | -4.1±2.6 | -4.6±2.6 | 0.690 | -4.0±2.3 | -4.3±2.6 | -4.6±2.9 | 0.645 |
| rs484066 (*ABCB11*) | -4.4±3.2 | -4.4±3.0 | -4.2±2.4 | 0.926 | -3.7±2.0 | -4.0±2.4 | -4.8±2.9 | 0.191 |
| rs569805 (*ABCB11*) | -4.4±3.3 | -4.5±3.0 | -4.2±2.4 | 0.890 | -3.8±2.2 | -3.8±2.3 | -4.8±2.8 | 0.156 |
| rs494874 (*ABCB11*) | -4.3±3.6 | -4.6±2.9 | -4.1±2.5 | 0.738 | -3.8±2.2 | -3.8±2.4 | -4.8±2.8 | 0.181 |
| rs2943641 (*IRS1*) | -3.1±2.0 | -4.6±2.9 | -4.4±2.8 | 0.402 | -4.1±2.0 | -4.6±3.0 | -4.0±2.2 | 0.561 |
| rs10182181 (*ADCY3*) | -4.2±3.5 | -4.4±2.4 | -4.4±3.2 | 0.951 | -4.1±2.2 | -4.2±2.9 | -4.6±2.0 | 0.756 |
| rs713586 (*ADCY3*) | -4.7±3.5 | -4.2±2.4 | -4.4±3.2 | 0.776 | -4.1±2.2 | -4.1±2.9 | -4.8±2.3 | 0.470 |
| rs2860323 (*TMEM18*) | -5.2±3.9 | -4.7±3.2 | -4.1±2.5 | 0.513 | -2.9 | -4.1±3.5 | -4.4±2.1 | 0.764 |
| rs2867125 (*TMEM18*) | -5.2±3.9 | -4.7±3.2 | -3.9±2.3 | 0.349 | -2.9 | -4.1±3.6 | -4.3±2.0 | 0.863 |
| rs13021737 (*TMEM18*) | -5.2±3.9 | -4.5±3.3 | -4.2±2.4 | 0.662 | -2.9 | -4.2±3.6 | -4.4±2.1 | 0.815 |
| rs1801282 (*PPARG*) | -4.2±2.6 | -5.7±3.7 | - | 0.074 | -4.4±2.6 | -3.8±2.3 | -4.0 | 0.713 |
| rs2959272 (*PPARG*) | -3.6±2.2 | -4.5±3.2 | -5.0±2.4 | 0.222 | -4.2±2.7 | -4.5±2.6 | -4.0±2.5 | 0.715 |
| rs1386835 (*PPARG*) | -4.5±2.6 | -4.2±3.1 | - | 0.677 | -4.3±2.4 | -4.6±2.9 | - | 0.545 |
| rs709158 (*PPARG*) | -4.6±2.7 | -4.4±3.0 | -3.3±2.2 | 0.397 | -4.2±2.5 | -4.5±2.8 | -3.2±1.5 | 0.319 |
| rs1175540 (*PPARG*) | -4.6±2.8 | -4.4±3.0 | -3.3±2.2 | 0.393 | -4.4±2.5 | -4.5±2.8 | -3.2±1.5 | 0.358 |
| rs1175544 (*PPARG*) | -4.5±2.8 | -4.5±3.0 | -3.3±2.2 | 0.403 | -4.3±2.6 | -4.5±2.8 | -3.4±1.7 | 0.386 |
| rs1797912 (*PPARG*) | -4.4±2.9 | -4.5±2.9 | -3.6±1.9 | 0.655 | -4.2±2.6 | -4.6±2.8 | -3.4±1.9 | 0.346 |
| rs1516725 (*ETV5*) | -1.4±1.9 | -4.1±1.9 | -4.5±2.9 | 0.269 | -3.3 | -4.2±2.4 | -4.3±2.7 | 0.909 |
| rs9816226 (*ETV5*) | -2.7±2.1 | -5.1±3.1 | -4.3±2.7 | 0.236 | -4.8±2.1 | -4.1±2.4 | -4.3±2.7 | 0.902 |
| rs13107325 (*SLC39A8*) | -4.5±2.8 | -3.1±2.7 | - | 0.169 | -4.2±2.7 | -4.7±1.8 | - | 0.426 |
| rs1799883 (*FABP2*) | -3.9±2.2 | -4.3±3.1 | -4.5±2.7 | 0.841 | -3.2±3.1 | -4.4±2.7 | -4.4±2.4 | 0.475 |
| rs6536991 (*UCP1*) | -4.5±2.8 | -4.1±2.7 | -3.7±3.0 | 0.668 | -4.3±2.4 | -3.9±2.7 | -5.2±3.0 | 0.347 |
| rs12502572 (*UCP1*) | -4.4±2.7 | -4.5±2.9 | -3.1±3.1 | 0.531 | -4.4±2.4 | -4.0±2.7 | -4.7±3.0 | 0.590 |
| rs1800592 (*UCP1*) | -4.6±2.8 | -4.0±2.9 | -3.1±2.1 | 0.506 | -4.2±2.6 | -4.7±2.6 | -2.2±2.6 | 0.329 |
| rs8192678 (*PPARGC1A*) | -4.5±2.4 | -4.2±3.4 | -4.6±2.1 | 0.878 | -4.3±2.4 | -4.1±2.9 | -4.6±2.6 | 0.866 |
| rs10938397 (*GNPDA2*) | -3.6±2.4 | -4.4±3.3 | -4.5±2.2 | 0.581 | -4.9±3.5 | -4.4±2.4 | -3.6±2.3 | 0.279 |
| rs1801260 (*CLOCK*) | -4.1±2.6 | -4.3±2.9 | -6.6±3.5 | 0.120 | -4.3±2.5 | -4.5±2.8 | -3.5±2.4 | 0.540 |
| rs1440581 (*PPM1K*) | -4.7±2.3 | -4.4±3.1 | -3.9±2.8 | 0.625 | -4.6±3.6 | -4.4±2.4 | -4.0±2.5 | 0.712 |
| rs1042713 (*ADRB2*) | -4.1±2.5 | -4.7±3.1 | -3.1±2.4 | 0.293 | -4.1±2.6 | -4.6±2.4 | -3.4±3.0 | 0.300 |
| rs1042714 (*ADRB2*) | -4.8±2.6 | -4.3±2.9 | -4.0±2.8 | 0.795 | -4.1±2.6 | -4.4±2.6 | -3.8±3.1 | 0.845 |
| rs6861681 (*CPEB4*) | -4.2±2.8 | -4.6±2.9 | -4.2±2.4 | 0.771 | -4.3±2.7 | -4.2±2.3 | -4.7±3.1 | 0.885 |
| rs1800629 (*TNFA*) | -4.3±2.6 | -4.2±3.2 | -11.0 | 0.056 | -4.3±2.7 | -4.5±2.4 | -3.0 | 0.830 |
| rs206936 (*NUDT3*) | -4.1±2.6 | -4.8±3.1 | -4.1±3.0 | 0.530 | -4.4±2.6 | -4.2±2.7 | -4.1±1.8 | 0.924 |
| rs987237 (*TFAP2B*) | -4.2±2.9 | -4.8±2.5 | -3.2±2.1 | 0.531 | -4.1±2.7 | -4.6±2.2 | -6.5±4.0 | 0.330 |
| rs2207139 (*TFAP2B*) | -4.2±2.9 | -4.8±2.6 | -3.2±2.1 | 0.502 | -4.1±2.6 | -4.9±2.5 | -3.7 | 0.372 |
| rs7799039 (*LEP*) | -3.8±3.0 | -4.3±2.6 | -4.7±2.9 | 0.543 | -4.4±2.5 | -4.2±2.7 | -4.4±2.5 | 0.877 |
| rs4731426 (*LEP*) | -4.0±2.9 | -4.3±3.0 | -4.5±2.6 | 0.859 | -4.5±2.4 | -4.0±2.8 | -4.5±2.3 | 0.606 |
| rs2071045 (*LEP*) | -4.4±2.8 | -4.3±2.8 | -3.9±2.8 | 0.934 | -4.2±2.6 | -4.4±2.7 | -4.1±2.3 | 0.912 |
| rs1055144 (*NFE2L3*) | -4.6±2.9 | -3.8±2.5 | -6.5 | 0.325 | -4.4±2.6 | -3.8±2.6 | -6.6±1.7 | 0.243 |
| rs4994 (*ADRB3*) | -4.3±2.6 | -4.6±3.5 | - | 0.695 | -4.5±2.7 | -3.4±1.7 | - | 0.146 |
| rs1800544 (*ADRA2A*) | -5.4±3.1 | -4.0±2.3 | -4.5±3.1 | 0.318 | -5.2±3.4 | -4.5±2.4 | -4.0±2.6 | 0.328 |
| rs2419621 (*ACSL5*) | -4.4±2.5 | -4.6±3.2 | -2.1±1.8 | 0.228 | -4.2±2.4 | -4.3±2.8 | -4.5±3.0 | 0.973 |
| rs7903146 (*TCF7L2*) | -3.7±2.3 | -4.4±3.1 | -5.6±2.4 | 0.076 | -4.7±2.8 | -4.1±2.3 | -3.4±2.7 | 0.326 |
| rs12255372 (*TCF7L2*) | -3.7±2.5 | -4.5±2.9 | -5.0±2.8 | 0.193 | -4.6±2.8 | -4.0±2.4 | -3.8±2.4 | 0.423 |
| rs1800497 (*ANKK1*) | -4.4±2.9 | -4.5±2.6 | -2.8±2.0 | 0.513 | -4.2±2.5 | -4.7±2.9 | -3.7±2.4 | 0.620 |
| rs662799 (*APOA5*) | - | -3.9±3.0 | -4.4±2.8 | 0.529 | -8.5 | -3.3±2.5 | -4.4±2.6 | 0.069 |
| rs6265 (*BDNF*) | -3.9±2.5 | -5.3±3.1 | -4.9±4.0 | 0.071 | -4.2±2.4 | -4.3±3.0 | -6.0±1.5 | 0.479 |
| rs11030104 (*BDNF*) | -3.8±2.4 | -5.4±3.2 | -4.7±3.1 | **0.036** | -4.2±2.4 | -4.4±2.9 | -4.7±3.0 | 0.858 |
| rs10767664 (*BDNF*) | -4.0±2.8 | -5.7±3.3 | -3.7±2.3 | **0.008** | -4.5±2.6 | -4.6±2.9 | -4.0±2.4 | 0.583 |
| rs11605924 (*CRY2*) | -4.2±2.0 | -4.3±2.9 | -4.7±3.6 | 0.817 | -4.0±2.4 | -4.5±2.9 | -4.2±2.1 | 0.756 |
| rs10838738 (*MTCH2*) | -4.3±2.8 | -4.4±3.0 | -4.3±2.5 | 0.984 | -4.9±2.5 | -4.1±2.4 | -3.6±2.9 | 0.147 |
| rs660339 (*UCP2*) | -4.7±2.6 | -4.2±3.0 | -3.7±2.7 | 0.508 | -4.0±2.7 | -4.8±2.6 | -3.3±1.8 | 0.090 |
| rs659366 (*UCP2*) | -5.0±3.0 | -3.9±2.5 | -3.5±2.6 | 0.109 | -4.1±2.7 | -4.7±2.7 | -3.4±1.8 | 0.215 |
| rs2075577 (*UCP3*) | -4.7±3.4 | -4.3±2.6 | -4.2±2.7 | 0.767 | -4.5±2.5 | -4.3±2.4 | -4.1±3.0 | 0.880 |
| rs2734827 (*UCP3*) | -5.0±3.0 | -3.8±2.5 | -3.8±2.8 | 0.094 | -4.4±2.3 | -4.3±2.9 | -3.9±2.4 | 0.839 |
| rs1685325 (*UCP3*) | -4.4±3.1 | -4.4±2.7 | -4.2±2.8 | 0.952 | -4.4±2.3 | -4.3±2.6 | -4.1±2.9 | 0.926 |
| rs2075576 (*UCP3*) | -4.4±2.9 | -4.6±2.6 | -2.3±2.0 | 0.286 | -4.4±2.6 | -4.1±2.7 | -3.6±1.5 | 0.743 |
| rs1800006 (*UCP3*) | -4.3±2.9 | -4.6±2.6 | -2.3±2.0 | 0.282 | -4.4±2.6 | -4.0±2.7 | -4.2±2.3 | 0.782 |
| rs1800849 (*UCP3*) | -4.4±2.9 | -4.6±2.6 | -2.3±2.0 | 0.286 | -4.3±2.6 | -4.2±2.7 | -3.6±1.5 | 0.836 |
| rs4929949 (*STK33*) | -4.5±3.0 | -4.8±2.7 | -3.4±2.6 | 0.115 | -4.7±3.2 | -4.4±2.4 | -3.8±2.3 | 0.451 |
| rs10830963 (*MTNR1B*) | -4.4±2.8 | -4.3±2.8 | -4.6±3.0 | 0.943 | -4.5±2.7 | -4.3±2.5 | -3.3±2.4 | 0.488 |
| rs4769873 (*ALOX5AP*) | -4.1±2.7 | -4.9±3.0 | -7.2±3.5 | 0.204 | -4.4±2.6 | -3.7±2.3 | -2.5 | 0.424 |
| rs1052700 (*PLIN1*) | -4.0±3.0 | -4.5±2.7 | -5.3±2.4 | 0.336 | -4.2±2.6 | -4.6±2.6 | -3.0±2.5 | 0.274 |
| rs894160 (*PLIN1*) | -4.7±2.9 | -4.0±2.5 | -3.5±3.2 | 0.347 | -4.5±2.9 | -4.2±2.1 | -1.9±1.2 | 0.091 |
| rs2289487 (*PLIN1*) | -4.6±3.2 | -4.1±2.7 | -4.4±2.8 | 0.788 | -3.0±3.1 | -4.2±1.8 | -4.6±2.9 | 0.218 |
| rs7498665 (*SH2B1*) | -4.2±3.0 | -4.5±2.6 | -4.6±2.4 | 0.881 | -4.5±2.9 | -4.3±2.1 | -3.7±2.7 | 0.610 |
| rs7359397 (*SH2B1*) | -4.2±3.0 | -4.4±2.5 | -4.5±2.5 | 0.936 | -4.4±2.9 | -4.3±2.1 | -3.7±2.7 | 0.659 |
| rs1558902 (*FTO*) | -4.5±3.0 | -3.9±2.5 | -5.1±3.1 | 0.367 | -4.4±2.6 | -4.2±2.6 | -4.4±2.7 | 0.877 |
| rs1121980 (*FTO*) | -4.5±2.9 | -3.8±2.5 | -5.1±2.9 | 0.246 | -4.5±2.7 | -4.3±2.6 | -4.0±2.6 | 0.830 |
| rs17817449 (*FTO*) | -4.5±3.0 | -4.2±2.6 | -4.6±3.0 | 0.828 | -4.5±2.6 | -4.3±2.6 | -3.5±2.1 | 0.525 |
| rs8050136 (*FTO*) | -4.5±3.0 | -4.2±2.6 | -4.6±3.0 | 0.828 | -4.5±2.6 | -4.3±2.6 | -3.5±2.1 | 0.525 |
| rs3751812 (*FTO*) | -4.5±3.0 | -4.2±2.6 | -4.6±3.1 | 0.852 | -4.5±2.6 | -4.3±2.7 | -3.5±2.1 | 0.546 |
| rs9939609 (*FTO*) | -4.5±3.0 | -4.1±2.6 | -4.6±2.9 | 0.813 | -4.5±2.6 | -4.3±2.6 | -3.5±2.1 | 0.525 |
| rs12452844 (*AANAT*) | -4.6±2.9 | -4.3±2.7 | -2.2±0.70 | 0.435 | -4.3±2.3 | -4.2±2.9 | -4.1±3.2 | 0.967 |
| rs1805081 (*NPC1*) | -4.5±2.7 | -4.2±2.8 | -4.3±2.5 | 0.835 | -4.5±2.9 | -4.6±2.5 | -3.2±1.6 | 0.127 |
| rs6567160 (*MC4R*) | -4.6±3.0 | -3.9±2.4 | -4.0±2.0 | 0.497 | -4.4±2.7 | -3.9±2.4 | -5.1±2.2 | 0.470 |
| rs571312 (*MC4R*) | -4.6±3.0 | -3.9±2.4 | -4.0±2.0 | 0.497 | -4.4±2.7 | -4.0±2.4 | -5.1±2.2 | 0.528 |
| rs17782313 (*MC4R*) | -4.4±2.8 | -5.0±1.3 | -4.2 | 0.929 | -4.2±2.6 | -7.7±1.5 | -4.4±2.2 | 0.065 |
| rs17066866 (*MC4R*) | -4.3±2.8 | -6.1±2.7 | - | 0.266 | -4.3±2.6 | -4.7±2.1 | - | 0.782 |
| rs17069904 (*TNFRSF11A*) | -4.5±3.0 | -3.8±2.1 | - | 0.356 | -4.0±2.3 | -5.4±3.4 | -6.4 | 0.088 |
| rs2287019 (*QPCTL*) | -4.4±3.0 | -4.0±2.5 | - | 0.583 | -4.1±2.5 | -4.8±2.7 | - | 0.173 |
| rs6013029 (*CTNNBL1*) | -4.3±2.6 | -4.5±3.7 | - | 0.847 | -4.3±2.7 | -4.3±2.0 | -4.2 | 0.999 |
| rs6123837 (*GNAS*) | -4.4±3.1 | -4.3±2.5 | -4.3±3.2 | 0.996 | -4.2±3.0 | -4.3±2.4 | -4.3±2.2 | 0.962 |
| rs3813929 (*HTR2C*) | -4.5±2.8 | -3.2±2.7 | -5.2±2.4 | 0.182 | -4.7±2.6 | -2.6±1.7 | -4.4±2.4 | **0.008** |
| rs11091046 (*AGTR2*) | -4.0±3.3 | -4.3±2.7 | -4.7±2.8 | 0.702 | -4.7±2.7 | -3.4±2.1 | -4.8±2.9 | **0.029** |

Comparison of means between genotypes by ANOVA tests. Variables are expressed as means ± SD. MHP: moderately high-protein; LF: low-fat. Bold numbers indicate statistically significant. 0 = wild-type homozygotes; 1 = heterozygotes; 2 = mutant homozygotes.

**Supplementary Table 4a.** Multiple linear regression models using genetic, phenotypic, and environment information to explain WCR as dependent variable in the MHP diet.

| Predictors | LARS | BSRP (adj. R^2^) | BSRP (AIC and AICC) | BSRP (BIC) and BSM | uGRS | **wGRS** |
| --- | --- | --- | --- | --- | --- | --- |
| Baseline WC (cm) | -0.073 | -0.073 | -0.079 | - | -0.082 | **-0.067** |
| Age (years) | -0.056 | -0.056 | - | - | - | **-0.052** |
| Sex | - | - | - | - | - | **-** |
| Baseline physical activity (METs) | - | - | - | - | - | **-** |
| Baseline energy intake (100 kcal) | -0.045 | - | - | - | -0.083 | **-** |
| rs2605100 (*LYPLAL1*) | 2.226 | 2.284 | 2.599 | 2.872 | - | **-** |
| rs662799 (*APOA5*) | -1.641 | -1.713 | -1.654 | - | - | **-** |
| rs1685325 (*UCP3*) | 1.975 | 1.985 | 2.074 | - | - | **-** |
| rs1558902 (*FTO*) | 2.044 | 2.024 | - | - | - | **-** |
| rs1121980 (*FTO*) | 1.979 | 2.109 | 2.755 | 2.569 | - | **-** |
| rs3813929 (*HTR2C*) | 2.477 | 2.635 | 2.428 | 3.422 | - | **-** |
| uGRS | - | - | - | - | 1.366 | **-** |
| wGRS | - | - | - | - | - | **0.959** |
| Constant | -1.149 | -2.562 | -3.241 | -11.629 | -1.390 | **-3.304** |
| Optimism-corrected adj. R^2^ | 0.0620 | 0.0812 | 0.1016 | 0.0911 | 0.0997 | **0.2081** |

Data are expressed as β values. Bold column indicate best model. Hyphens represent “not significant” or “not applicable” variables within each model. METs: metabolic equivalents; uGRS: unweighted genetic risk score; wGRS: weighted genetic risk score; LARS: least-angle regression; BSRP: best subset regression procedure; BSM: bootstrapping stepwise method; AIC: akaike information criterion; AICC: corrected akaike information criterion; BIC: bayesian information criterion; WCR: waist circumference reduction; MHP: moderately high-protein.

**Supplementary Table 4b.** Multiple linear regression models using genetic, phenotypic, and environment information to explain TFATL as dependent variable in the MHP diet.

| Predictors | LARS | BSRP (adj. R^2^) | BSRP (AIC y AICC) | BSRP (BIC) | BSM | uGRS | **wGRS** |
| --- | --- | --- | --- | --- | --- | --- | --- |
| Baseline TFAT (%) | 0.158 | 0.154 | - | - | - | 0.135 | **0.125** |
| Age (years) | -0.041 | -0.041 | -0.051 | -0.052 | -0.045 | -0.053 | **-0.043** |
| Sex | -3.495 | -3.382 | - | - | - | - | **-** |
| Baseline physical activity (METs) | - | - | - | - | - | - | **-** |
| Baseline energy intake (100 kcal) | - | - | - | - | - | - | **-** |
| rs2605100 (*LYPLAL1*) | 0.797 | 0.783 | - | - | 0.816 | - | **-** |
| rs3123554 (*CNR2*) | 0.949 | 0.963 | 1.159 | - | 0.955 | - | **-** |
| rs1801282 (*PPARG*) | -1.315 | -1.511 | -7.334 | -6.615 | -6.578 | - | **-** |
| rs7903146 (*TCF7L2*) | 0.763 | 0.908 | 1.255 | - | - | - | **-** |
| rs12255372 (*TCF7L2*) | 0.359 | - | - | - | - | - | **-** |
| rs6265 (*BDNF*) | - | - | - | - | - | - | **-** |
| rs11030104 (*BDNF*) | - | - | - | - | - | - | **-** |
| rs10767664 (*BDNF*) | 1.318 | 1.367 | 1.488 | 1.712 | 1.565 | - | **-** |
| rs659366 (*UCP2*) | 0.638 | 0.651 | 0.969 | - | 0.962 | - | **-** |
| rs2734827 (*UCP3*) | 0.524 | 0.562 | - | - | - | - | **-** |
| rs3813929 (*HTR2C*) | 1.173 | 1.131 | - | - | - | - | **-** |
| rs1801282 (*PPARG*) x sex | 3.601 | 3.508 | - | - | - | - | **-** |
| rs1801282 (*PPARG*) x baseline TFAT (%) | -0.006 | - | 0.201 | 0.194 | 0.192 | - | **-** |
| uGRS | - | - | - | - | - | 0.610 | **-** |
| wGRS | - | - | - | - | - | - | **0.915** |
| Constant | -10.587 | -10.543 | -5.857 | -4.537 | -5.843 | -11.269 | **-12.092** |
| Optimism-corrected adj. R^2^ | 0.0973 | 0.1552 | 0.2119 | 0.1813 | 0.2011 | 0.2810 | **0.3208** |

Data are expressed as β values. Bold column indicate best model. Hyphens represent “not significant” or “not applicable” variables within each model. METs: metabolic equivalents; uGRS: unweighted genetic risk score; wGRS: weighted genetic risk score; LARS: least-angle regression; BSRP: best subset regression procedure; BSM: bootstrapping stepwise method; AIC: akaike information criterion; AICC: corrected akaike information criterion; BIC: bayesian information criterion; TFATL: total body fat loss; MHP: moderately high-protein.

**Supplementary Table 4c.** Multiple linear regression models using genetic, phenotypic, and environment information to explain WCR as dependent variable in the LF diet.

| Predictors | LARS | BSRP (adj. R^2^) | BSRP (AIC and AICC) | BSRP (BIC) | BSM | **uGRS** | wGRS |
| --- | --- | --- | --- | --- | --- | --- | --- |
| Baseline WC (cm) | - | - | - | - | - | **-** | - |
| Age (years) | - | 0.110 | - | - | 0.126 | **-** | - |
| Sex | 1.214 | 1.049 | 1.548 | - | - | **1.636** | 1.575 |
| Baseline physical activity (METs) | - | - | - | - | - | **0.182** | 0.163 |
| Baseline energy intake (100 kcal) | -0.102 | -0.110 | -0.108 | -0.120 | -0.124 | **-0.083** | -0.084 |
| rs10838738 (*MTCH2*) | 1.012 | 7.806 | - | - | 9.519 | **-** | - |
| rs17069904 (*TNFRSF11A*) | 2.415 | 2.246 | 2.348 | 2.260 | - | **-** | - |
| rs11091046 (*AGTR2*) | 1.797 | 1.511 | 1.746 | 2.334 | 1.858 | **-** | - |
| rs10838738 (*MTCH2*) x age (years) | - | -0.141 | - | - | -0.174 | **-** | - |
| uGRS | - | - | - | - | - | **3.250** | - |
| uGRS x baseline physical activity (METs) | - | - | - | - | - | **-0.081** | - |
| wGRS | - | - | - | - | - | **-** | 1.381 |
| wGRS x baseline physical activity (METs) | - | - | - | - | - | **-** | -0.032 |
| Constant | -11.272 | -15.962 | -10.592 | -9.505 | -14.062 | **-15.425** | -15.175 |
| Optimism-corrected adj. R^2^ | 0.1564 | 0.1415 | 0.1657 | 0.1620 | 0.1308 | **0.2208** | 0.2106 |

Data are expressed as β values. Bold column indicate best model. Hyphens represent “not significant” or “not applicable” variables within each model. METs: metabolic equivalents; uGRS: unweighted genetic risk score; wGRS: weighted genetic risk score; LARS: least-angle regression; BSRP: best subset regression procedure; BSM: bootstrapping stepwise method; AIC: akaike information criterion; AICC: corrected akaike information criterion; BIC: bayesian information criterion; WCR: waist circumference reduction; LF: low-fat.

**Supplementary Table 4d.** Multiple linear regression models using genetic, phenotypic, and environment information to explain TFATL as dependent variable in the LF diet.

| Predictors | LARS | BSRP (adj. R^2^) | BSRP (AIC and AICC) | BSRP (BIC) | BSM | uGRS | **wGRS** |
| --- | --- | --- | --- | --- | --- | --- | --- |
| Baseline TFAT (%) | 0.147 | 0.189 | 0.162 | 0.180 | 0.162 | - | **0.194** |
| Age (years) | -0.00012 | - | - | - | - | - | **-** |
| Sex | 0.259 | 0.157 | 0.345 | - | 0.345 | 1.184 | **-** |
| Baseline physical activity METs | -0.010 | - | - | - | - | - | **-** |
| Baseline energy intake (100 kcal) | -0.038 | -0.036 | -0.037 | - | -0.037 | -0.139 | **-0.107** |
| rs484066 (*ABCB11*) | 1.912 | 2.070 | 2.093 | 1.051 | 2.093 | - | **-** |
| rs660339 (*UCP2*) | 0.197 | - | - | - | - | - | **-** |
| rs1805081 (*NPC1*) | 0.506 | - | - | - | - | - | **-** |
| rs17069904 (*TNFRSF11A*) | 1.350 | 1.170 | 1.382 | 1.381 | 1.382 | - | **-** |
| rs2287019 (*QPCTL*) | 0.994 | 1.020 | 1.013 | - | 1.013 | - | **-** |
| rs3813929 (*HTR2C*) | 1.642 | 9.837 | 1.574 | - | 1.574 | - | **-** |
| rs11091046 (*AGTR2*) | 12.112 | 11.627 | 11.870 | 15.127 | 11.870 | - | **-** |
| rs484066 (*ABCB11*) x sex | -1.732 | -1.731 | -1.796 | - | -1.796 | - | **-** |
| rs3813929 (*HTR2C*) x baseline TFAT (%) | - | -0.181 | - | - | - | - | **-** |
| rs11091046 (*AGTR2*) x baseline TFAT (%) | -0.247 | -0.242 | -0.242 | -0.318 | -0.242 | - | **-** |
| uGRS | - | - | - | - | - | -0.050 | **-** |
| uGRS x baseline energy intake (100 kcal) | - | - | - | - | - | 0.032 | **-** |
| wGRS | - | - | - | - | - | - | **2.490** |
| wGRS x baseline energy intake (100 kcal) | - | - | - | - | - | - | **0.020** |
| wGRS x sex | - | - | - | - | - | - | **-** |
| wGRS x baseline TFAT (%) | - | - | - | - | - | - | **-0.052** |
| Constant | -12.314 | -13.975 | -13.243 | -13.851 | -13.243 | -3.847 | **-12.130** |
| Optimism-corrected adj. R^2^ | 0.2157 | 0.2772 | 0.2858 | 0.2618 | 0.2858 | 0.3326 | **0.3792** |

Data are expressed as β values. Bold column indicate best model. Hyphens represent “not significant” or “not applicable” variables within each model. METs: metabolic equivalents; uGRS: unweighted genetic risk score; wGRS: weighted genetic risk score; LARS: least-angle regression; BSRP: best subset regression procedure; BSM: bootstrapping stepwise method; AIC: akaike information criterion; AICC: corrected akaike information criterion; BIC: bayesian information criterion; TFATL: total body fat loss; LF: low-fat.


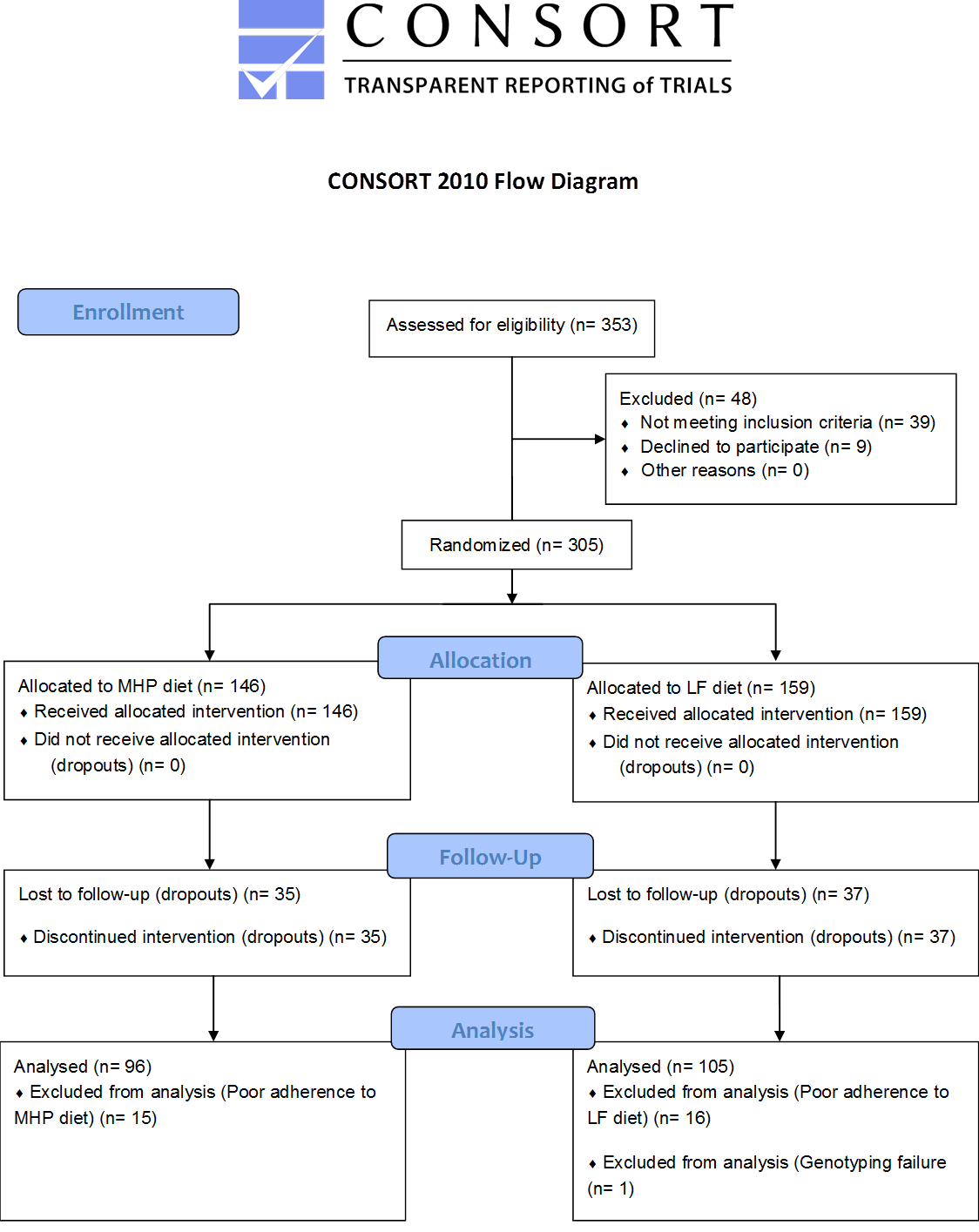


**Supplementary Figure 1.** Study design and flow of participants throughout the trial according to the 2010 CONSORT requirements.
